# Discovery and mapping of genomic regions governing economically important traits of Basmati rice

**DOI:** 10.1186/s12870-015-0575-5

**Published:** 2015-08-21

**Authors:** Lakshminarayana R Vemireddy, Sabahat Noor, VV Satyavathi, A Srividhya, A Kaliappan, SRN Parimala, Prathibha M Bharathi, Dondapati A Deborah, KV Sudhakar Rao, N Shobharani, EA Siddiq, Javaregowda Nagaraju

**Affiliations:** Institute of Biotechnology, Acharya NG Ranga Agricultural University, Rajendranagar, Hyderabad, 500030 AP India; Centre for DNA Fingerprinting and Diagnostics, Hyderabad, 500001 India; Indian Institute of Rice Research, Hyderabad, India

**Keywords:** Basmati rice, Quantitative trait loci, Quality traits, Microsatellite markers, Non-synonymous SNPs, Candidate genes

## Abstract

**Background:**

Basmati rice, originated in the foothills of Himalayas, commands a premium price in the domestic and international markets on account of its unique quality traits. The complex genetic nature of unique traits of Basmati as well as tedious screening methodologies involved in quality testing have been serious constraints to breeding quality Basmati. In the present study, we made an attempt to identify the genomic regions governing unique traits of Basmati rice.

**Results:**

A total of 34 Quantitative Trait Loci (QTLs) for 16 economically important traits of Basmati rice were identified employing F_2_, F_3_ and Recombinant Inbred Line (RIL) mapping populations derived from a cross between Basmati370 (traditional Basmati) and Jaya (semi-dwarf rice). Out of which, 12 QTLs contributing to more than 15 % phenotypic variance were identified and considered as major effect QTLs. Four major effect QTLs coincide with the already known genes viz., *sd1, GS3, alk1* and *fgr* governing plant height, grain size, alkali spreading value and aroma, respectively. For the remaining major QTLs, candidate genes were predicted as *auxin response factor* for filled grains, *soluble starch synthase 3* for chalkiness and *VQ domain containing protein* for grain breadth and grain weight QTLs, based on the presence of non-synonymous single nucleotide polymorphism (SNPs) that were identified by comparing Basmati genome sequence with that of Nipponbare.

**Conclusions:**

To the best of our knowledge, the current study is the first attempt ever made to carry out genome-wide mapping for the dissection of the genetic basis of economically important traits of Basmati rice. The promising QTLs controlling important traits in Basmati rice, identified in this study, can be used as candidates for future marker-assisted breeding.

**Electronic supplementary material:**

The online version of this article (doi:10.1186/s12870-015-0575-5) contains supplementary material, which is available to authorized users.

## Background

Rice, a staple food for over half of the global population, is endowed with rich genetic diversity, which is evident from the availability of numerous landraces and improved cultivars in the gene banks. Basmati is a unique varietal group of rice germplasm that has gained popularity as a speciality rice worldwide, mainly due to conscious and continuous selection by man over thousands of years for his diverse quality preferences [1].

Basmati rice occupies a special place among all aromatic rice cultivars by virtue of its unique quality characterized by extra long slender grain, lengthwise excessive kernel elongation upon cooking, soft and fluffy texture of the cooked rice, and exquisite aroma. It is, therefore, regarded as the “King of rices” [2–4]. Furthermore, previous diversity studies of rice revealed that the Basmati rice forms a separate cluster quite apart from *indica* and *japonica* groups [3, 5, 6]. Basmati expresses its unique features only when grown in the North-Western foothills of the Himalayas. Due to its location specific quality performance, Basmati is now a Geographical Indication (GI) in the Indian subcontinent. India has exported 3.75 Million MT of Basmati Rice to the world for the worth of USD 4,865 million during the year 2013–14 (www.apeda.gov.in).

In order to develop rice varieties suitable to various consumer quality preferences, knowledge of the genetics of key quality traits is inevitable. In the past, several genes/QTLs governing quality traits were identified in *indica* and *japonica* sub species of *Oryza sativa*. The major genes related to quality traits includes *waxy* gene for amylose content (AC) [7], *alk* gene for gelatinization temperature (GT) [8], *fgr* for fragrance [9, 10], *GS3* for grain size and grain weight [11] and *chalk5* for chalkiness [12]. In addition to these major genes, there are many minor QTLs governing the traits in *japonica* [13, 14] and *indica* [15]. Although a vast literature is available on the genetics and mapping of QTLs in *indica* and *japonica* rice varieties, not much information is available on Basmati rice *per se*. Among the limited number of studies available, one QTL for kernel elongation after cooking has been identified on chromosome 8 employing two RFLP markers viz., RZ323 and RZ562 [16]. Four QTLs for amylose content, two for gel consistency (GC) and five for gelatinization temperature (GT) have been identified from a cross between jasmine variety KDML105 and non aromatic CT9933 [17]. Using bulked segregant analysis of 247 F_2_ individuals of a cross between Basmati370/ASD 16, two microsatellite markers RM225 and RM247 have been identified and reported to be associated with grain breadth and cooked grain breadth, respectively [18]. Subsequently, QTLs for grain length (L), grain breadth (B), LB ratio, aroma, kernel elongation ratio, amylose content and alkali spreading value have been identified in a mapping population derived from a cross between Pusa1121, an evolved Basmati cultivar and Pusa1342 [19].

The aim of the present study was to identify and map QTLs linked to economically important traits of Basmati rice. Also, an attempt has been made to discover the candidate genes underlying the major QTLs by aligning Basmati genome sequence with available Nipponbare rice genome sequence information.

## Methods

### Plant Materials

**Fig. 1:**
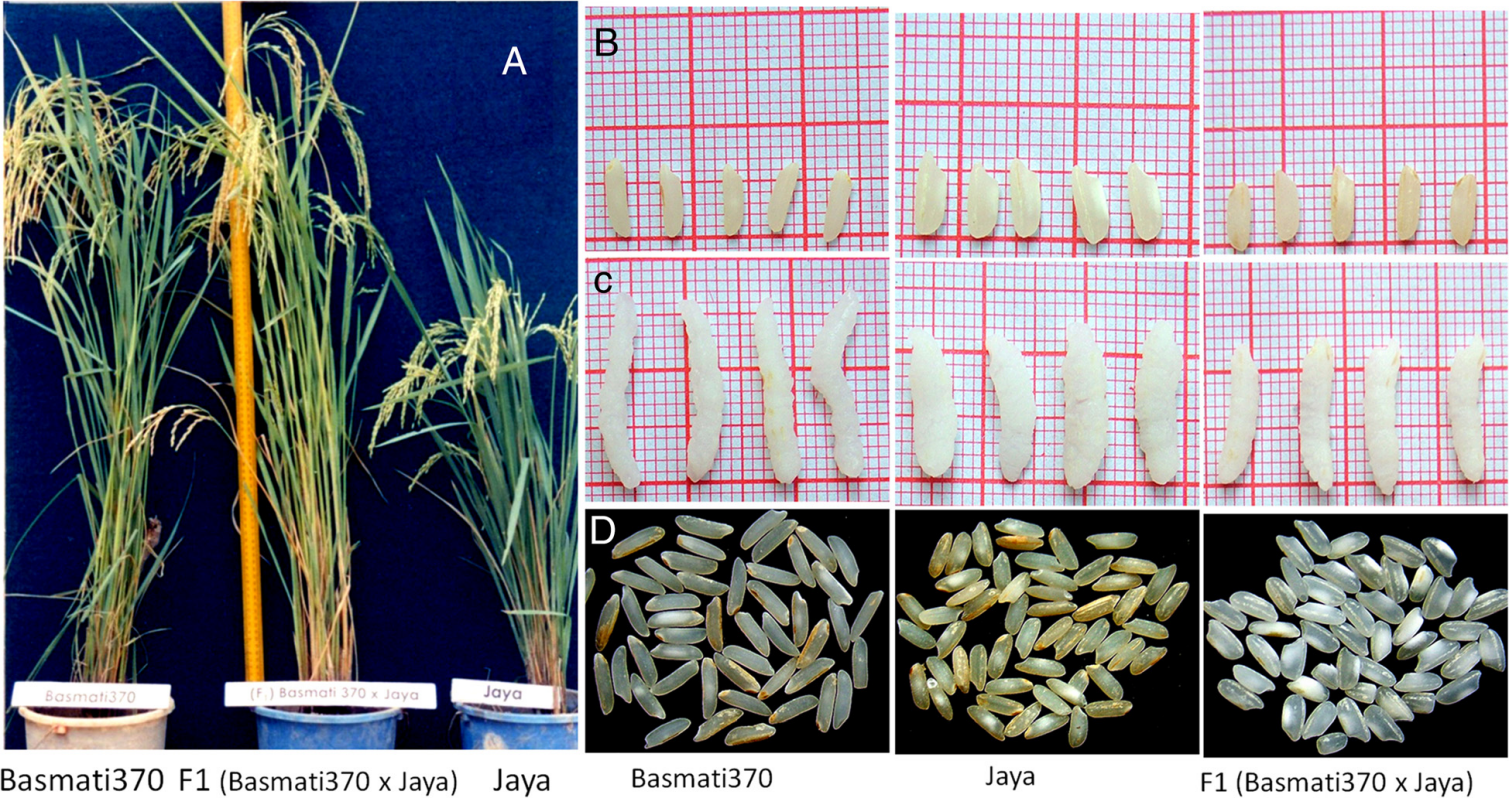
Agronomic and quality traits of Basmati370, Jaya and F_1_. **a**. Plant phenotypes of Basmati370, F_1_ and Jaya; **b** - **c**. Grain appearance traits of Basmati370, Jaya and F_1_ before and cooking and F_1_ before and cooking respectively; **d**. Grain chalkiness of Basmati370, Jaya and F_1_

The traditional Basmati variety, Basmati370 and the semidwarf non‐Basmati variety, Jaya were chosen as parents for developing a mapping population for the following reasons. The traditional Basmati varieties known by different names in the subcontinent, in all likelihood, are derivatives of the single local variety i.e., Basmati370 or Basmati370‐like variety [3]. Most of the Basmati varieties released as elite Basmati varieties since 1965 from India (12 of 19) and Pakistan (4 of 5) have Basmati370 as one of the donor strains in the breeding programs. Genetic diversity study employing ISSRs (Inter Simple Sequence Repeats) and SSRs (Simple Sequence Repeats) reveals that the high yielding variety Jaya to be genetically quite distinct from Basmati370 [3]. The parents Jaya and Basmati varieties possess distinct and contrasting physico‐chemical characters especially Jaya has very high amylose content than Basmati370. The genetic material consisted of progenies derived from a cross between Basmati370 and Jaya. One hundred F_1_ seeds were used to raise F_2_ generation during *Kharif,* 2005. The plant phenotype, grain appearance before and after cooking, and chalkiness characters of Basmati370, Jaya and their F_1_ hybrid and F_2_ progeny are shown in Fig. 1; Additional file 1: Figure S1. The F_2_ population was grown along with F_1_s and the parents in wet land farm of the Agricultural Research Institute (ARI), Rajendranagar, Hyderabad. Out of 10,000 F_2_ plants, 181 were randomly chosen as mapping population for construction of the linkage map and QTL mapping. The F_2_ population was advanced to F_3_ for the validation of the QTLs identified in the F_2_ population. To confirm the inheritance of the agronomic traits, one more set of F_2_ population comprising of 282 plants of the same cross was grown in Andhra Pradesh Rice Research Institute (APRRI), Maruteru, West Godavari, AP. In addition, a total of 155 recombinant inbred lines (RILs) was developed from the F_2_ individuals by single-seed descent method and grown in *kharif* 2009. The phenotypic measurements were recorded using the standard procedures for the eighteen traits in the mapping populations as given below.

*Plant height (PH)* - Length of the tallest tiller from ground level to the tip of the panicle, *Number of panicles (NP)* - Number of ear bearing tillers per plant, *Panicle length (PL)* - Length in cm from neck to the tip of the panicle excluding awn, *Spikelet number (SN)* - Number of spikelets including empty and filled ones per panicle averaged over 4–5 panicles, *Filled grains (FG)* - Number of filled spikelets per panicle averaged over 4–5 panicles, *Chaffy grains (CG)* - Number of sterile spikelets or chaffy grains per panicle averaged over 4–5 panicles, *Spikelet fertility (SF)* - Ratio of filled spikelets to the total number of filled and chaffy spikelets per panicle, expressed in percentage, *Grain weight (GW)* - Weight in grams of 1000 filled spikelets, *Single plant yield (SPY)* - Weight in grams of total filled grains per plant.

After maturation, the grains were harvested and stored at room temperature for at least 3 months before processing. The analysis of quality traits was carried out at Directorate of Rice Research (DRR), Hyderabad. Hulls were removed from 50 g of rough rice from each plant using a Huller (Model TH035A Satake, Houston, TX) to obtain brown rice. Embryos and the bran layers were removed (polished) from brown rice using miller (McGill, Model 1, Phillip Rahm International). The standard procedures were followed for recording data of quality traits as mentioned below:

*Grain length (GL) and grain breadth (GB)* - Measured using grain shape tester or dial micrometer for a minimum of 10 full rice grains with both the tips intact, *Grain length- breadth (LB ratio) -* Calculated as the grain length divided by grain breadth, *Chalkiness -* Ten whole grains from each of the plant were placed on light box for scoring chalkiness. Degree of chalkiness was determined by adopting the Standard Evaluation System for Rice, IRRI-2002 protocols, *Grain length after elongation (GLAC) and elongation ratio (ER) -* Kernels of rice varieties expand either breadth wise or lengthwise upon cooking. The elongation test consisted of soaking of 25 whole milled kernels in 20 ml of distilled water for 10 minutes and subsequently placing them in water bath at 98 °C for 10 min. The cooked rice was then transferred to a Petri dish lined with filter paper. Ten cooked whole grains were selected and length was measured by placing them on graph paper. The elongation was measured as the ratio of the average length of cooked rice kernels to the average length of uncooked rice kernels, *Aroma -* The presence of aroma from the rice leaf was evaluated by following the method developed by Sood and Siddiq [20]. A strongly scented variety, Basmati370 and a non-scented variety Jaya were used as checks for scoring of aroma, *Alkali Spreading Value (ASV)/Gelatinization temperature (GT) -* The method of Little *et al*. [21] was used for conducting the alkali spreading test. A duplicate set of six whole-milled grains without cracks was selected and placed in a plastic box (5 cm × 5 cm × 1.9 cm) containing 1.7 % KOH solution at 29 °C for 23 hrs. Then grains were carefully separated using forceps, and ASV of the grains was scored by visual assessment by seven scale score following Standard Evaluation System for Rice, IRRI-2002 protocols, and *Amylose content (AC) -* The procedure of Juliano *et al.* [22] was used for estimation of AC.

### Phenotypic data analysis of parents, F_1_ and F_2_ individuals

Correlations between character pairs and test for normal distribution were computed at p <0.05 and p < 0.01 in Microsoft-Excel (2007). Heterosis, heterobeltiosis and inbreeding depression were calculated using the following formulae.

$$ \begin{array}{l}\mathrm{Heterosis} = \left[\left(\mathrm{F}1-\mathrm{M}\mathrm{P}\right)/\mathrm{M}\mathrm{P}\right]\ \mathrm{x}\ 100\\ {}\mathrm{Heterobeltiosis} = \left[\left(\mathrm{F}1-\mathrm{B}\mathrm{P}\right)/\mathrm{B}\mathrm{P}\right]\ \mathrm{x}\ 100\end{array} $$

Where, MP is Mid parent and BP is Better parent

$$ \mathrm{Inbreeding}\ \mathrm{depression} = \left[\left(\mathrm{F}1 - \mathrm{F}2\right)/\ \mathrm{F}1\right]\ \mathrm{x}\ 100 $$

Tests of significance among parents, F_1_ and mid parental values were calculated employing StatPlus v 4.6 software (www.analystsoft.com/en).

### Construction of SSR linkage map

DNA from leaf material of the parents i.e., Basmati370 and Jaya, F_1_, F_2_, F_3_ and recombinant inbred lines (RIL) was extracted by using the modified CTAB method [23]. PCR amplification was performed in a 10 μl volume containing 10 mM Tris–HCl (pH 8.3), 1.5 mM MgCl_2_, 0.5 unit of Taq polymerase, 50 μM of dNTPs, and 0.1 μM of each primer with 10 ng of genomic DNA on a Thermal Cycler (PE9700) with a Ramp speed of 9700 (Applied Biosystems, USA). PCR samples were mixed with bromo-phenol blue and run on a 3 % agarose gel (Sigma) containing ethidium bromide along with 50 bp ladder (MBI Fermentas). Gels were photographed using Bio-Rad Molecular Imager Gel Doc XR System.

A set of 552 SSR markers spanning all the 12 rice chromosomes was screened between Basmati370 and Jaya strains. Out of which, 134 markers that were polymorphic between parents were used for screening the populations. The heterozygosity of the F_1_ hybrids has been confirmed using the polymorphic markers. The χ2 goodness of fit against 1:2:1 segregation ratio in the F_2_ population was tested using MapDisto software [24]. Linkage map was constructed using the MAPMAKER version 3.0 [25] following Kosambi mapping function. Linkage groups were determined using 'group' command with LOD score of 3.0 and a recombination fraction of 0.4. Order of the markers for each group was determined using 'order' and 'ripple' commands. Linkage groups were assigned to the respective chromosomes based on the rice genetic maps developed at Cornell University [26].

### QTL analysis

QTLs were detected by interval and composite interval mapping methods of Windows QTL Cartographer v.2.5 software. Composite interval mapping was conducted using the default settings (e.g., Model 6, five cofactors selected automatically by forward regression with a 10-cM window) (http://statgen.ncsu.edu/qtlcart/cartographer.html).

### Basmati genome sequencing

Basmati370 rice DNA was sequenced on SOLiD 4 using mate pair library kit with the insert size of 1.5 kb to 2.5 kb. Raw data was generated in csfasta and qual files, and was used for further analysis. Using Lifescope v2.5.1 software, the files were converted into xsq file format. Reads in xsq were mapped against Nipponbare reference sequence of complete rice genome sequence from http://rice.plantbiology.msu.edu/. Alignment results were produced in BAM file format to detect variations by variant caller algorithm. For variant annotation SnpEff (http://snpeff.sourceforge.net/) tool was used.

## Results

### Phenotypic evaluations and correlations among traits

**Fig. 2:**
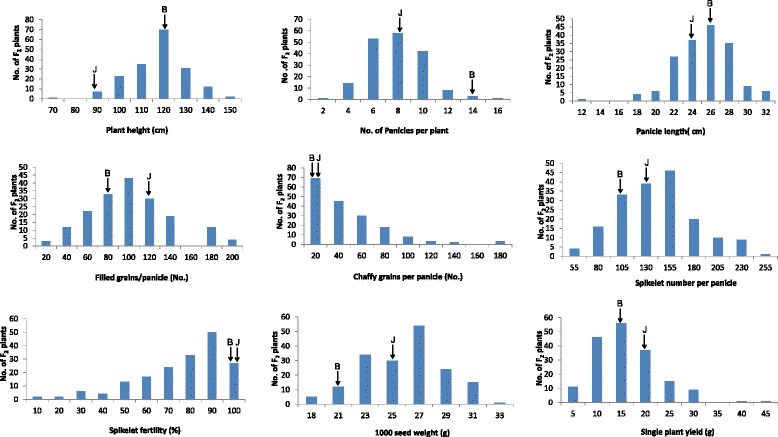
Phenotypic distributions of agronomic traits in 181 F_2 _ offspring derived from a cross between Basmati370 and Jaya. B - Basmati370; J - Jaya; F_1_ - Hybrid; F_2_ - F_2_​ progeny

**Fig. 3:**
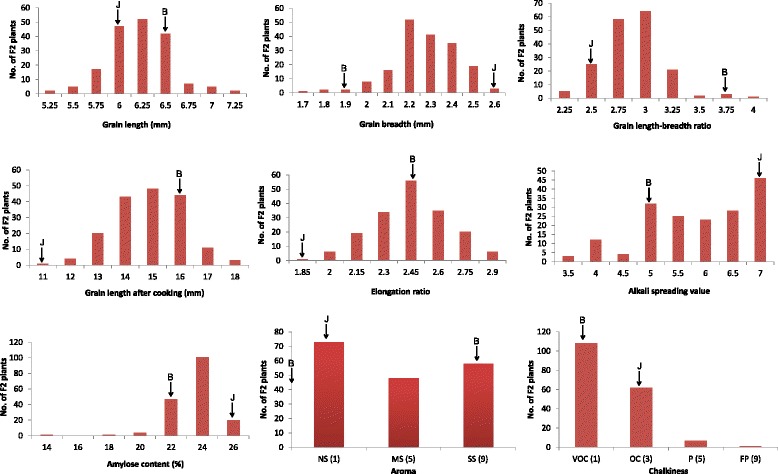
Phenotypic distributions of quality traits in 181 F_2 _ offspring derived from a cross between Basmati370 and Jaya. B - Basmati370; J- Jaya; F_1_ - Hybrid; F_2_ - F_2 _progeny

The parents Basmati370 and Jaya differed significantly (p < 0.05) with respect to majority of the traits studied, except for panicle length, chaffy grains, spikelet fertility and single plant yield (Fig. 1; Table 1). The mean of the F_1_ hybrids was intermediate for panicle length, 1000 seed weight, grain length (L), grain breadth (B) LB ratio, alkali spreading value, amylose content, and aroma. For rest of the traits, the F_1_ mean exceeded the mean of the better parent. Except aroma, all the agronomic and quality traits showed transgressive segregation ranging between 3 and 100 % (Figs. 2 & 3; Additional file 2: Table S1). As aroma is measured on 1–9 scale whereby the parents score the extremes of the scale, it was not possible to get transgressive segregants for this trait. However, in case of spikelet fertility, all the F_2_ plants fell below the parental average resulting in 100 % transgressive segregants. Transgressive segregants observed for the traits such as panicle length, filled grains, spikelet number, spikelet fertility, single plant yield and grain length significantly exceeded either of the parents. However, in case of plant height, grain length, elongation ratio, alkali spreading value and amylose content, transgressive segregants exceeded only Basmati370 whereas the number of panicles, chaffy grains and seed weight exceeded Jaya parent (Figs. 2 and 3; Additional file 3: Table S2). However, the number of transgressive segregants with respect to grain breadth, length-breadth ratio and chalkiness did not significantly (p > 0.05) exceed that of the parents.

**Table 1:** Test of significance among parents and F_1_s for 18 traits

S.No.	Trait	Code	Basmati370 (B)	Jaya (J)	F_1_	B/J
			(n = 10)	(n = 10)	(n = 10)	
1	Plant height (cm)	*PH*	114.79 ± 0.39	84.98 ± 4.65	120.25 ± 2.06	**
2	No. of panicles	*NP*	12.57 ± 3.64	8 ± 1.10	15 ± 2.94	*
3	Panicle length (cm)	*PL*	25.29 ± 2.66	23.33 ± 4.02	24.88 ± 1.03	NS
4	Filled grains (no.)	*FG*	75.50 ± 4.12	109.25 ± 4.65	167 ± 4.24	**
5	Chaffy grains (no.)	*CG*	4.86 ± 1.68	7.67 ± 4.50	20.50 ± 3.54	NS
6	Spikelet number	*SN*	80.25 ± 4.79	116.75 ± 0.50	187.5 ± 0.71	**
7	Spikelet fertility (%)	*SF*	94.13 ± 2.70	93.58 ± 4.09	89.06 ± 1.93	NS
8	1000 Seed weight (g)	*SW*	18.2 ± 2.27	23.65 ± 1.25	22.53 ± 1.49	**
9	Single plant yield (g)	*SPY*	14.19 ± 4.78	17.10 ± 1.10	27.96 ± 1.41	NS
10	Grain length (mm)	*GL*	6.49 ± 0.27	5.95 ± 0.37	6.24 ± 0.18	**
11	Grain breadth (mm)	*GB*	1.82 ± 0.05	2.53 ± 0.11	2.20 ± 0.05	**
12	Length-Breadth ratio	*LB*	3.57 ± 0.17	2.36 ± 0.18	2.84 ± 0.07	**
13	Grain length after cooking (mm)	*GLAC*	15.1 ± 0.57	9.88 ± 0.83	15.6 ± 0.84	**
14	Elongation ratio	*ER*	2.33 ± 0.17	1.68 ± 0.17	2.5 ± 0.15	**
15	Alkali spreading value	*ASV*	5 ± 0.00	7 ± 0.00	6.0 ± 1.05	**
16	Amylose content (%)	*AC*	21.03 ± 0.37	26.79 ± 0.29	22.8 ± 1.25	**
17	Aroma	*ARM*	9 ± 0.00	1 ± 0.00	2.00 ± 1.05	**
18	Chalkiness	*CHK*	1.80 ± 1.03	3 ± 1.63	1.60 ± 0.97	**

**Significant at p = 0.01 ; *Significant at p = 0.05; NS - Non-significant; n - Number of plants

Many of the quantitative traits showed normal distribution in F_2,_ F_3_ and RIL populations in both the environments (ARI, Hyderabad and APRRI, Maruteru) suggesting polygenic nature of the traits (Fig. 2; Additional files 4 and 5: Figures S2 & S3). As expected, in all the populations chaffy grains and spikelet fertility skewed towards the lowest and highest values, respectively. In contrast, amylose content and chalkiness showed unimodal distribution, whereas alkali spreading value, aroma and chalkiness showed abnormal distribution in F_2_ and RIL populations indicating that these traits might be under the control of few major genes and modifiers.

Of the agronomic traits, number of panicles and filled grains per panicle showed significant positive correlation with plant yield in F_2_ and RIL populations (Table 2; Additional file 6: Table S3). Spikelet number showed positive and significant correlation with panicle length, filled grains and chaffy grains (p < 0.05). Plant height also showed significant positive correlations with panicle length. As expected, spikelet fertility showed highly significant negative correlation with chaffy grains, while positive association with filled grains. Panicle length also showed a significant (p < 0.05) and positive association with filled grains and spikelet number.

**Table 2:** Correlation coefficients among 18 traits of F_2_ population derived from the cross between Basmati370 and Jaya

Trait	PH	NP	PL	FG	CG	SN	SF	SW	SPY	GL	GB	LB	GLAC	ER	ASV	AC	ARM	CHK
PH	1.000																	
NP	0.075	1.000																
PL	0.454*	−0.026	1.000															
FG	0.395	−0.006	0.557**	1.000														
CG	−0.046	0.054	0.090	−0.313	1.000													
SN	0.316	0.038	0.579**	0.643**	0.524**	1.000												
SF	0.208	−0.072	0.111	0.617**	−0.874**	−0.151	1.000											
SW	0.143	−0.059	0.158	0.066	0.132	0.166	−0.062	1.000										
SPY	0.345	0.629**	0.267	0.502**	−0.069	0.394	0.237	0.131	1.000									
GL	0.065	0.081	0.100	−0.033	0.032	−0.002	−0.029	0.223	0.129	1.000								
GB	0.063	0.020	0.051	0.038	0.177	0.172	−0.081	0.380	0.110	−0.266	1.000							
LB	−0.003	0.031	0.017	−0.024	−0.110	−0.106	0.052	−0.149	0.006	0.714**	−0.856**	1.000						
GLAC	0.039	0.165	0.145	0.261	0.008	0.238	0.065	0.227	0.245	0.402	−0.028	0.236	1.000					
ER	0.004	0.113	0.088	0.315	−0.014	0.268	0.092	0.097	0.174	−0.205	0.146	−0.203	0.811**	1.000				
ASV	−0.008	−0.114	0.181	0.009	0.009	0.014	0.019	0.029	−0.067	−0.074	−0.063	−0.008	−0.118	−0.082	1.000			
AC	0.123	−0.056	0.038	0.102	−0.188	−0.051	0.170	0.000	0.051	−0.030	−0.016	0.001	−0.076	−0.051	0.149	1.000		
ARM	0.130	0.115	0.063	−0.100	0.028	−0.07	−0.080	−0.082	0.100	0.099	−0.070	0.0979	0.034	−0.020	−0.030	−0.080	1.000	
CHK	−0.00	0.041	−0.040	0.057	0.020	0.066	−0.000	0.1844	0.119	−0.070	0.341	−0.27	0.129	0.173	−0.210	−0.060	−0.140	1.000

**Significant at p = 0.01 *Significant at p = 0.05 ; For trait codes refer Table 1

In case of quality traits, only grain appearance and cooking traits showed association in both the F_2_ and RIL populations. As expected, LB ratio showed a significant positive association with grain length and negative correlation with grain breadth. Similarly, grain length after cooking strongly associated with the elongation ratio (Table 2). The physico-chemical traits like amylose content, chalkiness, ASV did not show any association among themselves and with other traits clearly indicating the oligogenic nature of the traits.

**Table 3:** Comparison of Basmati genetic map with previously published rice genetic maps

	Current study	Qi-Jun *et al.* (2006) [35]	Temnykh *et al.* (2001) [36]	Harushima *et al.* (1998) [37]
Parents	Basmati370/Jaya	Nipponbare/93-11	IR64/Azucena	Nipponbare/Kasalath
Type of the population	F_2_	F_2_	DH	F_2_
Size of the population	181	90	96	186
Type of the markers	SSR	SSR	SSR & RFLP	RFLP
Number of the markers	134	152	>500 SSRs & 145 RFLPs	2275
Map length (cM)	2443.6	2455.7	1794.7	1521.6
Genetic distance between markers (cM)	18.23	16.16	2.78	<2
Physical distance between markers (kb)	3208.9	2828.9	666.7	189.01

### Parental polymorphism and segregation of marker loci

In the present study, 203 of the 552 microsatellite markers tested produced polymorphic and scorable bands (42.12 % polymorphism) between the parents Basmati370 and Jaya. Of 203 polymorphic loci, 60 markers which could not be scored were excluded from screening the F_2_ population. Nine markers were found to be unlinked. The remaining 134 markers used for construction of genetic linkage map comprised of 129 rice microsatellite markers, two from the waxy gene (MX4 and WXSSR), two markers linked to major QTL of grain length (RM353w and JL14), and one gene (*fgr*) specific STS (sequence tagged site) marker. Out of 134 markers, 98 (73.13 %) showed varying degrees of segregation distortion on all the 12 chromosomes suggesting that the distortion was random and not confined to any specific part of the rice genome (Additional file 3: Table S2). Majority of the markers represented heterozygotes, while very few (~9 %) showed Basmati370 alleles. The highest number of markers showing distorted segregation were mapped to chromosome 8 (12), whereas the lowest number (1) was mapped to chromosome 12.

### Linkage map

For mapping QTLs, a genetic map has been constructed employing 181 F_2_ offspring and 134 markers. The linkage map (LOD-score ≥3.0) placed 134 markers on 12 linkage groups spanning a total map length of 2443.6 cM with an average distance of 18.37 cM between adjacent marker loci. However, there were five large genetic gaps of 55–72 cM on chromosomes 1, 2, 8, 9 and 12. Excluding these genetic gaps, the average interval of remaining markers was 16.41 cM. A comparison of Basmati genetic map was made with previously published genetic maps and represented in Table 3.

### QTL Mapping

**Fig. 4:**
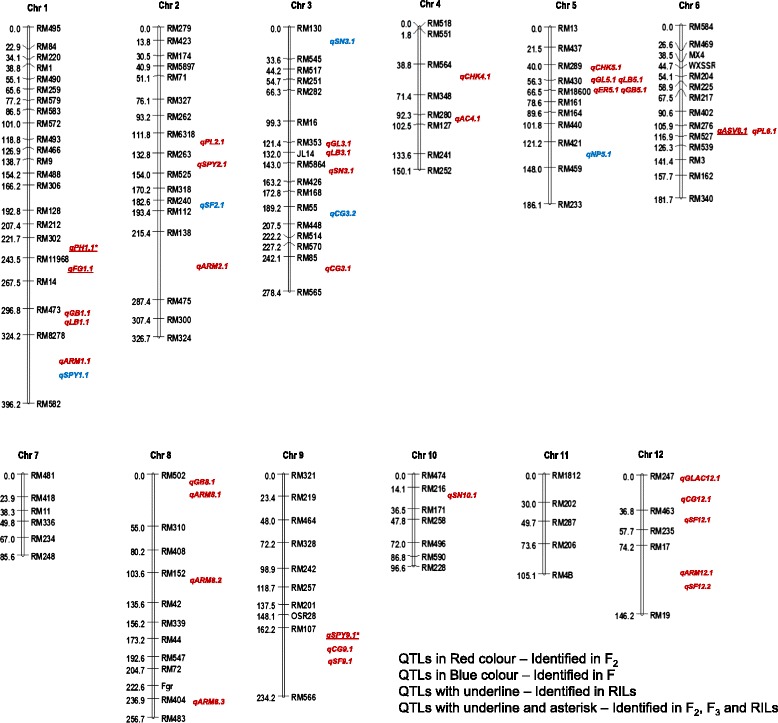
Distribution of QTLs for 16 traits in the molecular linkage map of Basmati. QTLs are indicated in bold (red colour) at right side of the linkage group. For codes of the traits refer Table 1. Names of the markers are represented left side of the linkage group. Numbers in parenthesis are genetic distance between markers in centimorgans (cM)

In all, 34 QTLs were identified for 16 agronomic and grain quality traits (Fig. 4;  Table 4). Of these, majority of the alleles with enhanced effect were found to be contributed by Basmati parent. Of 34 QTLs, 12 QTLs explained more than 15 % phenotypic variation between parents. Very few QTLs were identified for plant height, number of filled grains, spikelet number and single plant yield. This may be attributed to various reasons like genetically distant populations, non-detection of minor QTLs, and environmental effects.

**Table 4:** Quantitative trait loci (QTLs) detected in Basmati370/Jaya F_2_ population

SN	Trait	QTL	C	Marker interval	LFM	RFM	LOD	A	D	PVE
1	Plant height (cm)	*qPH1.1*	1	RM302-RM11968	16	10.4	5.138	7.908	−0.858	15.418
2	Panicle length (cm)	*qPL2.1*	2	RM6318-RM263	16	9.28	3.039	0.456	1.636	0.925
3		*qPL6.1*	6	RM276-RM527	2	10.22	3.413	0.408	−1.773	0.819
4	Filled grains (no.)	*qFG1.1*	1	RM11968-RM14	10	19.55	3.244	31.165	−28.073	22.677
5	Chaffy grains (no.)	*qCG3.1*	3	RM85-RM565	20	30.2	4.284	−3.532	−13.439	0.46
6		*qCG9.1*	9	RM107-RM566	34	80	3.021	−2.71	−10.48	0.328
7		*qCG12.1*	12	RM247-RM463	34	15.23	5.211	−7.804	−15.738	2.458
8	Spikelet number (no.)	*qSN3.1*	3	RM5864-RM426	14	10.16	2.788	666	−1.593	0
9		*qSN10.1*	10	RM216-RM171	26	1.29	2.885	−19.354	−6.115	6.661
10	Spikelet fertility (%)	*qSF9.1*	9	RM107-RM566	56	58	2.562	4.208	5.35	2.202
11		*qSF12.1*	12	RM463-RM235	14	11.15	7.255	7.155	1.973	4.249
12		*qSF12.2*	12	RM17-RM19	48	66	3.441	5.987	−4.491	4.472
13	Single plant yield (g)	*qSPY2.1*	2	RM263-RM525	0	25.55	3.72	−2.258	3.979	4.06
14		*qSPY9.1*	9	RM107-RM566	48	66	3.154	8.397	−4.769	8.15
15	Grain length (mm)	*qGL3.1*	3	RM353-JL14	10	1.7	9.217	0.362	−0.125	46.065
16		*qGL5.1*	5	RM430-RM18600	6	5.2	6.603	0.217	0.031	17.468
17	Grain breadth (mm)	*qGB1.1*	1	RM473A-RM8278	0	34.52	6.714	−0.038	0.119	1.649
18		*qGB5.1*	5	RM430-RM18600	4	7.2	3.333	−0.106	0.052	17.149
19		*qGB8.1*	8	RM502-RM310	16	64.66	3.454	666	0.015	0
20	Length-Breadth ratio	*qLB1.1*	1	RM473A-RM8278	0	34.52	5.063	0.116	−0.208	3.928
21		*qLB3.1*	3	RM353-JL14	8	3.7	4.358	0.22	−0.129	22.342
22		*qLB5.1*	5	RM430-RM18600	8	3.2	4.65	0.405	−0.07	46.531
23	Grain length after cooking (mm)	*qGLAC12.1*	12	RM247-RM463	0	49.23	3.512	0.312	0.396	2.68
24	Elongation ratio	*qER5.1*	5	RM430-RM18600	4	7.2	3.711	0.136	0.067	18.931
25	Alkali spreading value	*qASV6.1*	6	RM276-RM527	4	8.22	26.746	−1.257	0.264	71.735
26	Amylose content (%)	*qAC4.1*	4	RM280-RM127	0	11.15	4.077	−0.97	0.315	15.249
27	Aroma	*qARM1.1*	1	RM8278-RM582	74	40	6.735	0.654	5.284	1.859
28		*qARM2.1*	2	RM138-RM475	80	32.06	7.59	−0.178	−5.332	0.133
29		*qARM8.1*	8	RM502-RM310	36	44.66	6.976	−0.23	−5.309	0.218
30		*qARM8.2*	8	RM152-RM42	18	23.56	6.132	0.968	−5.312	3.116
31		*qARM8.3*	8	RM404-RM483	8	16	4.998	2.476	0.511	20.226
32		*qARM12.1*	12	RM17-RM19	30	84	7.556	−0.589	−5.334	1.512
33	Chalkiness	*qCHK4.1*	4	RM564-RM348	14	28.62	3.138	2.107	−0.142	63.795
34		*qCHK5.1*	5	RM289-RM430	6	12.88	3.835	−0.809	0.359	14.533

A- Additive; D- Dominance; C- Chromosome; PVE- Phenotypic variance explained by each QTL (%); Left (LFM) and right (RFM) flanking marker distance from the QTL (cM);Positive and negative values of additive effect indicates the increasing effect coming from the alleles of Basmati370 and Jaya, respectively.

#### QTLs for plant height

Only one QTL, designated as *qPH1.1*, was identified for plant height trait on chromosome 1 at an interval of RM302‐RM11968 and it accounted for 15.42 % phenotypic variance. Alleles from Basmati370 were associated with increased plant height.

#### QTLs for panicle length

Two minor QTLs were identified for panicle length. Of which, one QTL was on chromosome 2 (*qPL2.1*) and another on chromosome 6 (*qPL6.1*) with marker intervals of RM6318-RM263 and RM276-RM527, respectively. The enhanced quantitative effect was contributed by the Basmati370 suggesting that a major part of the variation in panicle length is due to environmental influence.

#### QTLs for filled grains

A single QTL designated as *qFG1.1* was identified on chromosome 1 in the marker interval of RM11968‐RM14. It explained 22.68 % of the phenotypic variance between the parents indicating the possible involvement of a major gene governing the trait. Increasing effect of this QTL resulted from the Basmati parent.

#### QTLs for chaffy grains

A total of three QTLs influencing chaffy grains designated as *qCG3.1, qCG9.1,* and *qCG12.1* were identified one each on chromosomes 1, 9 and 12, respectively. Together they explained 3.246 % phenotypic variation. The increasing effect at all the loci for chaffy grains was contributed by Jaya parent.

#### QTLs for spikelet number

Two regions were found to be associated with QTLs for spikelet number viz., *qSN3.1* and *qSN10.1* on chromosome 3 and 10, respectively. Of the two QTLs, the QTL *qSN3.1* explained zero percent phenotypic variation of the trait suggesting that the genes within this QTL region might be having opposite effects, whereas *qSN10.1* accounted for 6.7 % of the phenotypic variation with the allele from the Jaya parent contributing to the enhancing effect.

#### QTLs for spikelet fertility

Three QTLs, one on chromosomes 9 (*qSF9.1*) and remaining two on chromosome 12 (*qSF12.1* and *qSF12.2*) affecting spikelet fertility were identified. Together they accounted for 10.92 % of the phenotypic variance. At all the three loci Basmati parent contributed to spikelet fertility.

#### QTLs for single plant yield

Two QTLs, *qSPY2.1* and *qSPY9.1* were identified for single plant yield on chromosomes 2 and 9, respectively. The QTL *qSPY9.1* on chromosome 9 explained 8.15 % phenotypic variance. The other QTL, *qSPY2.1* accounted for only 4.06 % of the phenotypic variance. The allele for increased grain yield was contributed by Basmati370 for *qSPY9.1* and Jaya for *qSPY2.1*.

#### QTLs for grain length

A total of two QTLs viz., *qGL3.1* and *qGL5.1* with phenotypic variance of 46.01 % and 17.47 %, were detected on chromosomes 3 and 5, respectively. The increasing effect for these two QTLs was associated with Basmati370 allele.

#### QTLs for grain breadth

Three QTLs, *qGB1.1*, *qGB5.1* and *qGB8.1* were found to be responsible for grain breadth. Of them, one QTL, *qGB5.1* on chromosome 5 had a major effect explaining 17.15 % phenotypic variance and one QTL *qGB1.1* on chromosome 1 had a relatively minor effect explaining 1.65 % phenotypic variance. In all these QTLs, increased effect was contributed by the parent Jaya. For the QTL *qGB8.1*, Basmati370 and Jaya alleles have opposite effects resulting in zero percent variance in phenotype. The two QTLs, *qGB1.1* and *qGB8.1* identified in the present study appears to be novel.

#### QTLs for Length-Breadth ratio (LB)/Grain size

A total of three QTLs influencing this trait were identified. In all the QTLs, alleles from Basmati370 contributed to increase in LB ratio. The QTLs, *qLB3.1* on chromosome 3 and *qLB5.1* on chromosome 5 explained 22.34 and 46.53 % phenotypic variation, respectively. The other QTL, *qLB1.1* explained 3.93 % phenotypic variance.

#### QTLs for grain length after cooking (GLAC)

A QTL associated with GLAC, *qGLAC12.1* contributing 2.68 % phenotypic variance was located on chromosome 12. Basmati allele was associated with an increase of GLAC as was the case in grain length.

#### QTLs for elongation ratio (ER)

One QTL, *qER5.1* was identified for this trait on chromosome 5 explaining 18.9 % phenotypic variance. The allele from a Basmati370 contributed to the elongation ratio at this region.

#### QTLs for alkali spreading value (ASV)/ gelatinization temperature (GT)

**Fig. 5:**
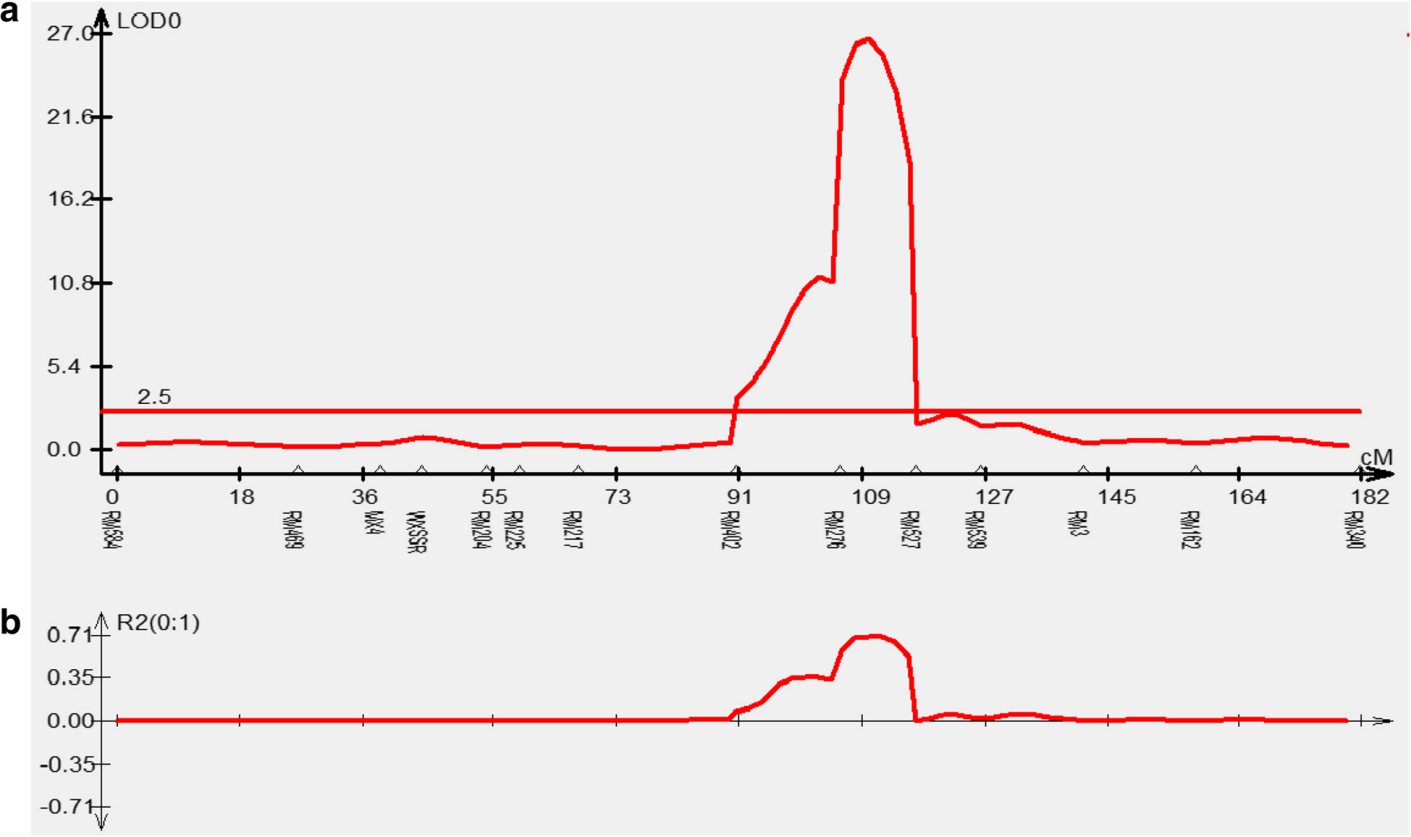
QTL cartographer LOD peak for alkali spreading value. **a**) Markers and their genetic distances are given in X-axis and LOD values in Y-axis; **b**) Phenotypic variance explained by the alkali spreading value QTL

One major QTL for ASV, *qASV6.1* on chromosome 6 was identified with the highest LOD value of 26.75 explaining a maximum of 71.74 % phenotypic variance. The allele from Jaya had a strong positive effect on ASV. QTL cartographer LOD peak for alkali spreading value is given in Fig. 5.

#### QTLs for amylose content (AC)

One QTL *qAC4.1* explaining 15.25 % phenotypic variance was detected on chromosome 4. The Jaya allele had an increasing effect on this trait. The QTL identified here is in contrary to the previous reports whereby the major QTL controlling AC (*waxy* gene) was located on chromosome 6.

#### QTLs for aroma

Six QTLs designated as *qARM1.1, qARM2.1, qARM8.1, qARM8.2, qARM8.3* and *qARM12.1* influencing aroma were identified. Of these, three QTLs *qARM8.1, qARM8.2* and *qARM8.3* were located on chromosome 8 explaining 0.22, 3.12, and 20.23 % phenotypic variance, respectively. The other three QTLs *qARM1.1, qARM2.1* and *qARM12.1* located on chromosomes 1, 2 and 12, respectively, together contributed 3.51 % phenotypic variance. These QTLs are novel ones and are specific to Basmati varieties as they are being reported for the first time.

#### QTLs for chalkiness (CHK)

A total of two QTLs, *qCHK4.1* and *qCHK5.1* were identified on chromosomes 4 and 5 with the increased effects from the Basmati and Jaya, respectively. The QTLs for grain breadth and chalkiness were found to be co-localised and showed a positive significant correlation. Our results are consistent with the earlier study [27].

### QTL clusters for grain appearance traits

In the present study, QTLs related to highly correlated traits like grain breadth, grain length, and length-breadth ratio were found to be located on the same genomic region of chromosome 5 *viz., qGL5.1, qGB5.2, qLB5.1* and *qER5.2* as reported earlier [15, 27, 28]. However, this trend was not seen for other significantly correlated traits such as plant height, panicle length, filled grains and single plant yield. The QTLs relating to these traits have been mapped onto different chromosomes implying that these traits are possibly controlled by independent and unrelated genes.

However, in the region of RM430 and RM18600 effects of three QTLs for grain breadth *(qGB5.2)*, grain length *(qGL5.1)*, and length-breadth ratio *(qLB5.1)* are in different directions, suggesting involvement of tightly linked genes as the cause of the correlation of these traits.

### Confirmation of QTLs in F_3_ population

As the quantitative traits are with low heritability, the phenotypic mean of the F_3_ progeny derived from each of the F_2_ plant along with its genotyping data was used (as was done earlier [29]) in order to confirm the mapped QTLs identified in F_2_ population. Using F_2:3_ design, we have identified a total of 10 QTLs for various agronomic traits viz., plant height (1), number of panicles (2), chaffy grains (2), spikelet number (1), spikelet fertility (1) and plant yield (3). Of these, two QTLs viz., *qPH1.1* for plant height and *qSPY9.1* for plant yield have been commonly observed in both F_2_ and F_2:3_ designs with a phenotypic variance of 21.55 % and 23.88 %, respectively (Additional file 7: Table S4).

### QTL mapping in the RIL population

When we compared the QTLs identified in the F_2_ population with that of RILs of the cross between Basmati370 and Jaya, we could identify only 12 common QTLs for 10 traits in both the populations (Additional file 8: Table S5). The phenotypic variance of all the QTLs except filled grains and plant yield was more than 15 % within a range of 9.3 to 73.52 %. In RIL population, QTL for alkali spreading value (*qASV6.1)* showed high LOD (27.33) and phenotypic variance (73.52 %) similar to that observed in the F_2_ population. This clearly suggests that even with preliminary mapping populations like F_2_, it is possible to identify the major QTLs with an appropriate population size.

### Gene ontology (GO) analysis of the genes underlying major QTLs

Since a typical QTL region contains several hundreds of genes, it is necessary to filter them further in order to pinpoint the right candidate gene(s) underlying the trait. Given the advances in rice genome annotation, now it is possible to integrate the putative gene function with the associated gene ontology (GO) terms. In the present study, the total number of genes underlying each major QTL interval was retrieved from the RiceTOGO Browser (http://agri-trait.dna.affrc.go.jp/). Using this list of total genes in each major-effect QTL marker interval, the percentage of annotated genes and significantly overrepresented GO terms were estimated. The percentage of annotated genes for each promising QTL varied from 84.56 % to 99.64 % with an average of 93.55 %, while significantly enriched or overrepresented GO terms ranged from zero to 17.42 %, the average being 4.41 % (Table 5).

**Table 5:** Known QTLs/ genes and GO terms underlying the major QTLs

Trait	Chr.	QTLs	Marker interval	Total no. of genes	No. gene annotated	Annotated genes (%)	No. significant GO terms	Significant GO terms (%	Known QTLs/Genes	Gene function
Plant Height	1	*qPH1.1*	RM302-RM11968	534	528	98.88	92	17.42	*sd1*	Gibberellin- 20 oxidase 2
Filled Grains	1	*qFG1.1*	RM11968-RM14	266	265	99.62	0	0.00		
Grain Length/LB Ratio	3	*qGL3.1/qLB3.1*	RM353-JL14	204	201	98.53	1	0.50	*qGL-3, kl3.1,qGL-3A, GS3, qLWR3*	GS3-Putative transmembrane protein
Grain Length/Breadth/LB Ratio	5	*qGL5.1/qGB5.1/qLB5.1/qER5.1*	RM430-RM18600	28	24	85.71	0	0.00		
Alkali Spreading Value	6	*qASV6.1*	RM276-RM527	242	209	86.36	0	0.00	*qGT-6*	soluble starch synthase II-3
Amylose content	4	*qAC4.1*	RM280-RM127	61	56	91.80	9	16.07		
Aroma	8	*qARM8.1*	RM404-RM483	88	87	98.86	0	0.00	Fgr	Betain aldehyde dehydrogenase-2
Chalkiness	4	*qCHK4.1*	RM564-RM348	1355	1181	87.16	52	4.40		

### Genomics based candidate genes prediction in the major QTL regions

In an attempt to identify the candidate genes for the novel major QTLs, we have sequenced the Basmati370 genome, compared with the publicly available Nipponbare sequence and shortlisted the genes with non-synonymous SNPs (nsSNPs). In the QTL interval governing the filled grain trait, we have identified 48/266 genes with nsSNPs within the targeted QTL regions. Previously, it has been demonstrated that the auxins have a role in the grain filling by regulating the invertase enzymes [30]. In the present study also, we have identified one auxin response factor (LOC_Os01g70270) found to have a nsSNP (cGa/cAa) in which arginine (R) was replaced by glutamine (Q) at position 530 (Additional file 9: Table S6). Transcriptome analysis by qTeller software (http://qteller.com/) provided further evidence that the expression of this gene is high at 25 days after pollination of the endosperm stage. Similarly, we were able to predict the candidate gene underlying the QTL cluster consisting of four QTLs viz., *qGL5.1, qGB5.1, qGLB5.1,* and *qER5.1* controlling grain appearance traits as VQ domain containing protein (LOC_Os05g32460) as it contains one nsSNP (aCt/aTt) where threonine was replaced by isoleucine.

## Discussion

With the advent of high yielding varieties ensuring higher farm returns, serious threat to Basmati rices was perceived by the breeders prompting them to resort to breeding for varieties of Basmati quality in high yielding background. But for reasons that are beginning to be understood, no variety ideally matching the traditional Basmati could be evolved. Genetic investigations have revealed that all traits except one or two are controlled quantitatively and selections based on phenotype are not reliable enough [19, 31]. The present study was undertaken with the objective of identifying QTLs governing the key characters of Basmati rice. We have identified 34 QTLs governing 16 economically important traits of Basmati rice employing F_2_, F_3_, and Recombinant Inbred Line (RIL) mapping population derived from a cross between Basmati370 and a semi-dwarf rice variety Jaya. Out of 12 major-effect QTLs identified, four QTLs coincided with the previously known genes *sd1, GS3, alk1* and *fgr* and for the remaining QTLs, candidate genes were predicted by comparing Basmati genome sequence with that of Nipponbare. So far, many major QTLs have been mapped in rice, however, to our knowledge, this study is the first attempt made to carry out genome-wide mapping for the dissection of the genetic basis of economically important traits of Basmati rice.

### Divergence and distinctness of Basmati rice

In the present study, the polymorphic markers were found distributed on all the 12 chromosomes of Basmati rice (Fig. 4). The existence of high parental polymorphism (42.12 %) provided evidence to the divergence and distinctness of Basmati rice from the other rice groups viz., *indica* and *japonica* [3, 32]. The percent polymorphism detected in this study is higher than the previously reported value (28.9 %) where an evolved Basmati variety (Pusa1121) was used [19] and lower (63.95 %) when a traditional Basmati (Basmati370) was used as a parent [18, 33]. The significant effects of distorted markers on linkage estimation provide insights for genetic mapping analysis of genes or QTLs. Out of 134 markers, 98 showed varying degrees of segregation distortion on all the 12 chromosomes suggesting that the distortion was random and not confined to any specific region of the rice genome (Additional file 3: Table S2). Our results are in agreement with earlier findings [19] wherein segregation distorted loci were distributed over eight chromosomes *viz.,* 2, 3, 4, 6, 7, 8, 9 and 10. Majority of the markers represented heterozygosity, while very few (~9 %) showed Basmati370 alleles. The highest number of markers (12) showing distorted segregation were mapped to chromosome 8, whereas the lowest number (1) was mapped to chromosome 12.

### Construction of linkage map

According to Lander and Botstein [34], the linkage map with an average interval less than 20 cM is suitable for QTL mapping. The genetic map of Basmati is 2443.6  cM and is shorter (2455.7 cM) than the map reported by Qi-Jun *et al.* [35] and longer than some of the notable maps constructed using inter-sub specific rice populations that are either 1794.7 cM [36] or 1521.6 cM [37] (Table 3).

In this study, we observed higher genetic distances between some of the markers and this could be attributed to (a) deviation of 73.13 % of markers from actual segregation ratios as pointed out in the previous study [38], (b) stretching effect of markers on chromosomes caused by small population size [38], and (c) map expansion due to excess heterozygosity in segregating markers. Our results are in agreement with that of Knox and Ellis [39]. The increase in the total map length due to stretching effect has been reported in several crops including rice [38], sorghum [40] and barley [41].

### QTL mapping of agronomic traits

Using populations derived from a cross between Basmati370 and Jaya parents, we detected 34 QTLs and compared them with previously reported ones. For plant height, only one QTL, designated as *qPH1.1*, was identified on chromosome 1. Interestingly, near *qPH1.1*, semi dwarf gene, *sd1* which encodes a *gibberellic acid 20-oxidase* (*OsGA20ox-1*) (*LOC_Os01g66100*), that catalyzes the conversion of GA_53_ to GA_20_ in gibberellic acid biosynthesis in rice [42–44] was found to be present. Ishimaru et al. (2004) identified a *sucrose phosphate synthase* gene controlling plant height on a different region of the same chromosome [45]. For panicle length two minor QTLs were identified one each on chromosome 2 (*qPL2.1*) and chromosome 6 (*qPL6.1*). Previous studies reported an *aberrant panicle organization-1 (APO-1)* gene encoding an F-box protein on chromosome 6. A mutation in this gene was reported to result in reduced panicle length and less number of spikelets per panicle [46]. In the present study we identified a single QTL designated as *qFG1.1* for filled grains on chromosome 1. Like plant height QTL, this QTL also was very close to *sd1* gene (~80 kb). The cytokinin accumulation in inflorescence meristems was previously reported to down regulate *OsCKX2* which then results in increase in the reproductive organs causing enhanced grain yield [47]. A gene underlying grain number QTL, *Gn1a* encoding cytokinin oxidase/dehydrogenase (*OsCKX2*) that degrades phytohormone cytokinin has also been reported on chromosome 1. However, the QTL detected in the present study and *Gn1a* are not same suggesting that *qFG1.1* seems to harbour other candidate genes that control grain number through mechanism(s) that are yet to be elucidated. A gene underlying major QTL (*Ghd7*) which encodes a CCT domain protein has also been identified on chromosome 7 with a major effect on the number of grains per panicle and heading date [48].

For single plant yield, we identified two QTLs, *qSPY2.1* and *qSPY9.1* on chromosomes 2 and 9, respectively. Previous reports identified a yield improving QTL GY2-1 using the parents Dongxiong (a wild rice, *Oryza rufipogan* Griff.) and Guichao2 (*Oryza sativa* ssp indica) and located it on upstream of the QTL *qSPY2.1* on chromosome 2 [47, 49]. This QTL was governed by a *leucine rich repeat receptor kinase* gene cluster.

### QTL mapping of grain appearance traits

A total of two QTLs for grain length viz., *qGL3.1* and *qGL5.1* were detected on chromosomes 3 and 5, respectively. Interestingly, these regions coincide with major QTLs reported for grain size by numerous other studies carried across different environments and genetic backgrounds [11, 50–53]. Therefore, the present study tends to support the general conclusion made earlier [54] that a substantial proportion of QTLs affecting a trait particularly those having major effects can be identified under different environments. The major QTL i.e., *GS3* which controls both grain length and weight has been previously identified on chromosome 3 [11]. It has been dissected into a gene which encodes a putative PEPB (Phosphatidyl ethanolamine-binding protein)-like domain, a transmembrane region, a putative TNFR (tumor necrosis factor receptor) /NGFR (nerve growth factor receptor) family cysteine rich domain, and a VWFC (von willebrand factor type C) module. Comparative sequence analysis identified a non-sense mutation in the second exon of the putative *GS3* gene in all long-grain varieties when compared to small grain varieties. This mutation causes a 178 amino acid truncation in the C-terminal region of the predicted protein, suggesting that *GS3* may function as a negative regulator for grain size [11]. It can be inferred that the major QTL for grain length detected in this study on chromosome 3 is likely to be the same locus as the one reported by earlier studies [51, 55]. It is also interesting to note that the chromosome region of maize flanked by *umc164c* and *umc157* on chromosome 1 harbouring a QTL for kernel length is homologous to the short arm of the rice chromosome 3 suggesting the possibility of orthology between rice and maize genes governing kernel length in this region [56]. The QTL, *qGL5.1* identified in the present study also coincides with the earlier reports. Since the underlying gene has not been identified yet, this QTL could be a potential candidate for dissection.

For grain breadth, out of three QTLs *qGB1.1*, *qGB5.1* and *qGB8.1* identified in the present study*,* two QTLs, *qGB1.1* and *qGB8.1* appears to be novel since major QTL/gene (s) reported by other groups were located on chromosomes 2 and 5. A major QTL for grain width, i.e., *GW2* on chromosome 2, has been identified; which encodes a RING type protein with *E3 ubiquitin ligase* activity and is known to function in the ubiquitin-proteasome pathway [57]. Further, loss of *GW2* function, increases cell number resulting in a larger or wider spikelet hull and accelerated grain milk filling rate which consequently enhances grain width, weight and yield. Similarly, a QTL for grain width, i.e., *qGW5* on chromosome 5 had been delimited to 2,263 bp fragment of Kasalath genomic region [58]. Comparative analysis of Kasalath revealed that Nipponbare region harboured a 1212 bp deletion and several SNPs. A recent study in maize demonstrated that the grain width gene on chromosome 2 i.e., *GW2* has two orthologous duplicated genes *viz*., *ZmGW2-CHR4* and *ZmGW2-CHR5* with similar function of controlling the kernel size and weight even after crop diversification during evolution [59].

The co‐localization of QTLs for grain breadth and chalkiness as well as positive significant correlation between these two traits observed in the present study suggests that breeders can simultaneously improve these two traits. These results are consistent with the earlier study [27, 60] where QTLs for grain width and chalkiness were mapped at a marker interval of RG360 and C37349 on the same region of the chromosome 5. Recently, it has been reported that a gene influencing grain chalkiness i.e., *Chalk5* encodes a vacuolar H^+^-translocating pyrophosphatase [12]. This gene is located upstream of *qCHK5.1.* Interestingly, a gene governing vacuolar-processing enzyme (*LOC_Os04g45470*) was located within the QTL region of *qCHK4.1.* In the same QTL region, *soluble starch synthase 3* (*LOC_Os04g53310*), a key enzyme in the starch biosynthesis pathway is also located. These two QTLs seem to be potential targets for manoeuvring chalkiness in rice.

A total of three QTLs influencing length-breadth ratio (LB) / grain size were identified, out of which *qLB3.1* on chromosome 3 and *qLB5.1* on chromosome 5 were located in the vicinities of *qGL3.1* controlling grain length and *qGB5.1* controlling grain breadth traits, respectively. Such association is not surprising because LB ratio is a derived trait obtained by dividing the grain length by grain breadth. Our results are consistent with previous reports obtained across different environments and genetic backgrounds [27, 28] suggesting that these QTLs are controlled by a few major genes with modifiers. Hence, these QTLs may be considered as potential candidates for future fine mapping and cloning studies.

### QTL mapping of cooking quality traits

We identified QTL for grain length after cooking (GLAC) on chromosome 12. Although the grain length after cooking is one of the unique quality traits of the Basmati rice, the genomic regions governing the trait are not yet identified. In non-Basmati rices, however, scattered reports of mapping QTL regions for this trait are available. Among them, initially, a QTL on chromosome 8 associated with cooked kernel elongation has been identified and concluded that this QTL was loosely linked to the fragrance gene [16, 61]. Subsequently, three QTLs on chromosomes 2, 6 and 11 [62] and a single QTL on chromosome 3 [56] and two QTLs each on chromosomes 2 and 6 [63] have been identified for this trait.

We have identified one QTL for elongation ratio (ER), *qER5.1* on chromosome 5. However, previously, a QTL for ER, *elr11-1* was identified on chromosome 11. Likewise, three more QTLs have been identified on chromosomes 2, 4 and 12 with major QTL being *qER-2* [64].

One major QTL for alkali spreading value, *qASV6.1* identified on chromosome 6 was mapped along with *alk* gene (Fig. 4). The *alk* gene encodes soluble *starch synthase IIa* (SSIIa) and is associated with gelatinization temperature. Thus our results are in agreement with the previous reports in showing that GT is primarily controlled by *alk* gene [17, 65, 66]. However, contrary to these results, it has been demonstrated that GT is controlled by a *wax*y gene [27, 67]. These observations infer that the genetic factors other than the *alk* gene are probably involved in altering the GT variation indicating that *alk* is a major but not the sole player in GT variation. Previous reports suggested that the SSIIa is one of the important biosynthetic enzymes determining starch structure and its properties [8, 68]. The SSIIa enzyme seems to have a role in the elongation of A and B1 amylopectin chains, and determines the ratio of two chain lengths, i.e., L- type (present in *indica* rices) and S-type (present in *japonica* rices) [8, 68]. However, in Basmati rice, being a separate group from *indica* and *japonica* rice, it would be interesting to understand the role as well as the structure of SSIIa. In the present study, we identified one QTL for *amylose content, qAC4.1* on chromosome 4. Although different amylose classes *viz.,* waxy (~0 %), low (2-19 %), intermediate (20-25 %) and high (>25 %) are known to be associated with the variability in the *waxy* gene which *encodes granule- bound starch synthase (GBSSI)* on chromosome 6, the *waxy* gene alone could not explain the global phenotypic variability of the trait due to the availability of subclasses within each major class prompting us to speculate the existence of the loci other than *waxy* gene [55]. Probably, the QTL identified in the present study interacts with the *waxy* locus to control the final amylose content which is specific to Basmati rices.

The key gene governing the aroma encodes *betain aldehyde dehydrogenase* (*badh2*) that is known to be located on chromosome 8. Further, it has been reported that all the fragrant rices harbour an 8 bp deletion when compared to the non-fragrant varieties [9]. We have identified six novel QTLs that are specific to Basmati variety as they are being reported for the first time. Contrary to many studies where aroma is reported to be controlled by a single recessive gene, in the present study aroma behaved like a polygenic trait. Of six QTLs for aroma, three from Basmati370 and four from Jaya explained the increased effect, suggesting that the environment where the experiment was conducted seemed to influence the expression of aroma. Moreover, Basmati needs cool temperatures during flowering period for expression of its unique traits especially pleasant aroma. The non-detection of major QTLs for the aroma could be attributed to the current experimental conditions.

### QTL clusters for grain appearance traits

Several earlier studies have demonstrated that QTLs for correlated traits often map to the same chromosome regions [29, 55, 69, 70]. In our study, we have found QTLs related to highly correlated traits like GB, GL and LB ratio to be located on the same genomic region of chromosome 5. Classical quantitative genetics assumes that trait correlation can be attributed to the effect of pleiotropy or to the tight linkage of causative genes. If pleiotropism is the major reason, coincidence of both the location of QTL for related traits as well as the direction of their genetic effects can be expected. If the tight physical linkage of the genes is the major reason, the direction of the genetic effect of QTL for different traits may be different, although the coincidence of the location of QTLs can still be expected [28].

### Stable QTLs or major QTLs of promise

The genomic regions or QTLs, which are consistently detected over a range of environments or mapping populations or different parental crosses, are considered “stable or major QTLs” and are preferred targets in crop improvement. Despite the fact that the present study was carried out by a single cross, the identified common QTLs in all the F_2_, F_3_ and RIL populations can be considered as stable or major effect QTLs. Together with the results of previous studies, seven QTLs viz., *qPH1.1* [42–44], *qGL3.1, qGB5.1, qLB3.1, qLB5.1* [11, 28]*, qASV6.1* [71] and *qARM8.2* [9, 72] that are associated with five traits of Basmati can be considered as stable QTLs. As described by Wan *et al*. [28], the major effect QTLs are more likely to behave as stable QTLs across multiple environments/genetic backgrounds. These QTLs, apart from their suitability in the improvement of the traits concerned, can also serve as potential candidates for fine mapping and also facilitate the development of near-isogenic lines and advanced breeding lines. Further, several QTLs, each with different environment specificity, can be introgressed into a single genotype to develop phenotypes stable over a range of environments. In fact, in conventional plant breeding, selections are made in target environment and testing is done in multiple diverse environments. This exercise is cumbersome and time consuming. However, use of stable QTLs based selection can accelerate the pace of selection process in rice breeding programs.

### Gene Ontology Analysis

The enriched GO terms and the likely candidate genes of each promising QTL have been studied. In the plant height QTL region flanked by the markers RM302 and RM11968, as many as 92 significant GO terms have been identified, of which, metabolic process (GO:0008152) and cellular process (GO:0044237) terms belonging to the class biological process of the gene ontology were overrepresented. Of the 92 GO terms identified, one gene corresponded to the well known Green revolution gene *sd1* (semi dwarfing) which also belongs to biological process class [73].

In case of grain length QTL on chromosome 3, only one significant GO term, i.e., caspase activity (GO:0030693) related to molecular function has been observed. This GO term corresponding to four genes, includes three ICE-like protease p20 domain containing proteins and one Zinc finger, LSD1-type domain containing protein. In this QTL region one major gene that codes for putative transmembrane protein (Os03g0407400) was found to be governing the grain length [11]. However, for this gene, no significant hit was available in the GO analysis.

In the genomic region governing amylose content, i.e., *qAC4.1,* nine significant GO terms have been identified. However, many of the genes belong to the DNA damage or repair mechanism. It may be presumed that these genes probably act as modifiers of the amylose content in addition to other known major genes like granule bound starch synthase (GBSS).

Even though, the region governing the chalkiness i.e., *qCHK4.1* is very large, only 52 significant GO terms were hit. Among them, metabolic process (GO:0008152), cell (GO:0005623) and catalytic activity (GO:0003824) are with the highest terms in the classes of biological process, cellular components and molecular function, respectively. A gene similar to *Chalk5* was found in the QTL region of *qCHK4.1* which belongs to the class of biological process and codes for vacuolar-processing enzyme (LOC_Os04g45470) [12]. However, in the same QTL region, soluble starch synthase 3 (LOC_Os04g53310) under the GO term of carbohydrate metabolic process also existed.

### Prediction of candidate genes in the major QTL regions of Basmati rice

Several recent publications indicate key intersecting signalling role for auxins and cell wall invertases (CWIN) during grain filling. [30]. In the present study, we have identified an *auxin response factor* (*LOC_Os01g70270*) found to have a nsSNP (cGa/cAa) in which arginine (R) was replaced by glutamine (Q) at position 530 using qTeller software (http://qteller.com/)(Additional file 9: Table S6).

We were also able to predict candidate gene underlying the QTL cluster consisting of four QTLs viz., *qGL5.1, qGB5.1, qGLB5.1,* and *qER5.1* controlling grain appearance trait as *VQ domain containing protein (LOC_Os05g32460)*. In *Arabidopsis*, the VQ motif protein *IKU1* has been reported to regulate endosperm growth and seed size along with *IKU1* and *MIN3* genes [73]. Similarly, based on the transcriptome analysis, *AP2 domain containing protein (LOC_Os05g32270)* and *RING E3 ligase (LOC_Os05g32570)* showing higher expression during early flowering stage were reported to be involved in regulating grain size in Arabidopsis by Ohto et al. [74] and in rice by Song et al. [57], respectively.

The enzyme involved in starch biosynthesis (*soluble starch synthase 3*) could be the plausible candidate gene for the chalkiness QTL region of RM564 and RM348 as it has been found to have one nsSNP (aaA/aaC) wherein lysine was replaced by asparagine at 207 position (Table 5; Additional file 10: Table S7). Interestingly, the same gene was overrepresented in our GO analysis as well, providing further evidence that this gene is a probable candidate for the chalkiness. However, its expression is less in the transcriptome analysis compared to the unknown genes.

## Conclusion

Basmati rice of the Indian subcontinent is a highly distinctive rice because of its unique grain quality, elongation upon cooking and aroma traits. With the advent of high yielding varieties ensuring high farm returns, serious threat to Basmati rices was perceived by the breeders pushing them to resort to breeding of varieties of Basmati quality in the high yielding background. However, no variety ideally matching the traditional Basmati quality could be evolved even after many decades of efforts. Genetic investigations have revealed that most of the Basmati-specific traits are controlled quantitatively and selections based on phenotype are not reliable enough. The present study was undertaken with the objective of identifying genomic regions or QTLs governing the key characters of Basmati rice using the cross between traditional Basmati variety, Basmati370 and high yielding non-Basmati variety Jaya. To the best of our knowledge, the current study is the first attempt to carry out combinational approach of genome-wide mapping and genomics assisted candidate gene prediction to dissect the genetic basis of important agronomic and quality traits of Basmati rice.

Molecular markers tightly linked to the stable and major QTLs can be of potential value in application of marker-assisted selection (MAS) of the corresponding traits in rice breeding. The major QTLs identified in the present study for economically important traits of Basmati can be transferred to high yielding varieties and parents of heterotic hybrids by recombination breeding using the tightly linked markers. Being a model cereal crop with all the available genetic and genomic resources, along with the basmati genomic sequence, the understanding of quality QTLs would facilitate their positional cloning. By pyramiding the genes from different varieties in a single variety it could be possible to develop a high yielding superior quality rice variety so that it can be available to the common man who dreams to taste speciality rices like Basmati.

## Additional files

Additional file 1: Figure S1. The grain appearance traits before and after cooking in the Basmati370, Jaya, F_1_ and selected F_2_ individuals. (TIFF 10085 kb) Additional file 2: Table S1. Transgressive segregants, heterosis, heterobeltiosis and inbreeding depression for 18 traits in the F_2_ population. (DOC 49 kb) Additional file 3: Table S2. Chi square values of microsatellite markers showing segregation distortion among F_2_ population of Basmati370/Jaya (DOC 154 kb) Additional file 4: Figure S2. Phenotypic distributions of agronomic and quality traits in RIL population derived from a cross between Basmati370 and Jaya. B - Basmati370; J- Jaya; F_1_: Hybrid. (TIFF 1004 kb) Additional file 5: Figure S3. Phenotypic distributions of agronomic traits in F_3_ population derived from a cross between Basmati370 and Jaya. B - Basmati370; J- Jaya; F_1_: Hybrid. (TIFF 2461 kb) Additional file 6: Table S3. Correlation coefficients among 18 traits of the RIL population derived from the cross of Basmati370 and Jaya. (DOC 60 kb) Additional file 7: Table S4. Quantitative trait loci (QTLs) detected in F_3_ population of Basmati370/Jaya. (DOC 35 kb) Additional file 8: Table S5. Quantitative trait loci (QTLs) detected in the RIL population derived from Basmati370/Jaya. (DOC 38 kb) Additional file 9: Table S6 The genes with non-synonymous SNPs in the QTL for filled grain qFG1.1. (RM11968-RM14). (DOC 65 kb) Additional file 10: Table S7 The genes with non-synonymous SNPs in the QTL for chalkiness qCHK4.1. (RM564-RM348) (DOC 168 kb)

## Abbreviations

cM: Centi Morgan
GO: Gene ontology
GT: Gelatinization temperature
KEGG: Kyoto Encyclopedia of Genes and Genomes
LB ratio: Length- Breadth ratio
LOD: Logarithm of odds ratio
MAS: Marker-assisted selection
nsSNPs: Non-synonymous SNPs
PCR: Polymerase chain reaction
QTL: Quantitative trait loci
RIL: Recombinant inbred line
