# Supplementary figures and images for: Discovery and mapping of genomic regions governing economically important traits of Basmati rice
Source: BMC Plant Biol. 2015 Aug 21;15:207. doi: 10.1186/s12870-015-0575-5 (PMC4546240; doi:10.1186/s12870-015-0575-5)

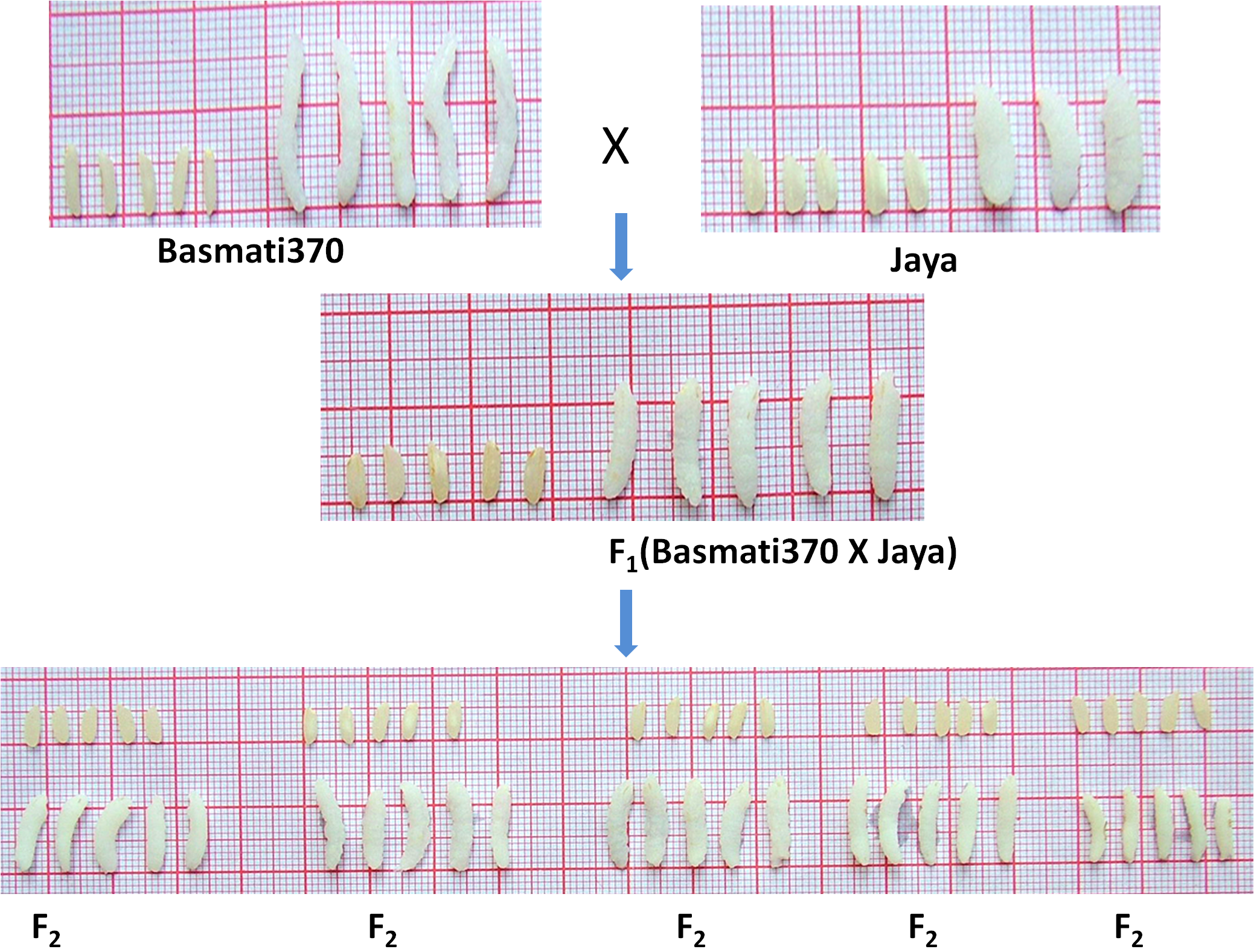

Supplement: Additional file 1: Figure S1. — The grain appearance traits before and after cooking in the Basmati370, Jaya, F1 and selected F2 individuals. (TIFF 10085 kb) [file 12870_2015_575_MOESM1_ESM.tif]

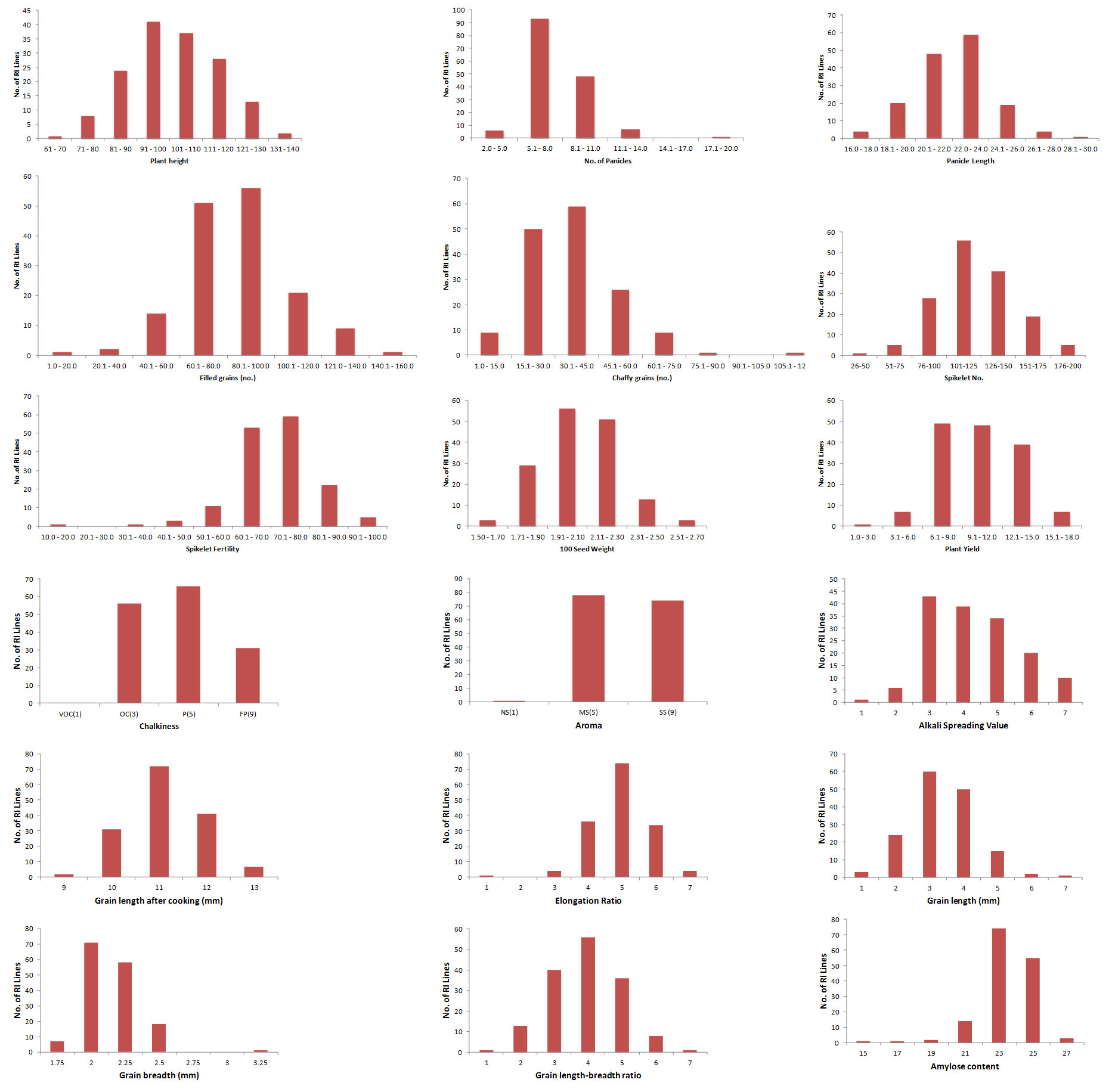

Supplement: Additional file 4: Figure S2. — Phenotypic distributions of agronomic and quality traits in RIL population derived from a cross between Basmati370 and Jaya. B - Basmati370; J- Jaya; F1: Hybrid. (TIFF 1004 kb) [file 12870_2015_575_MOESM4_ESM.tif]

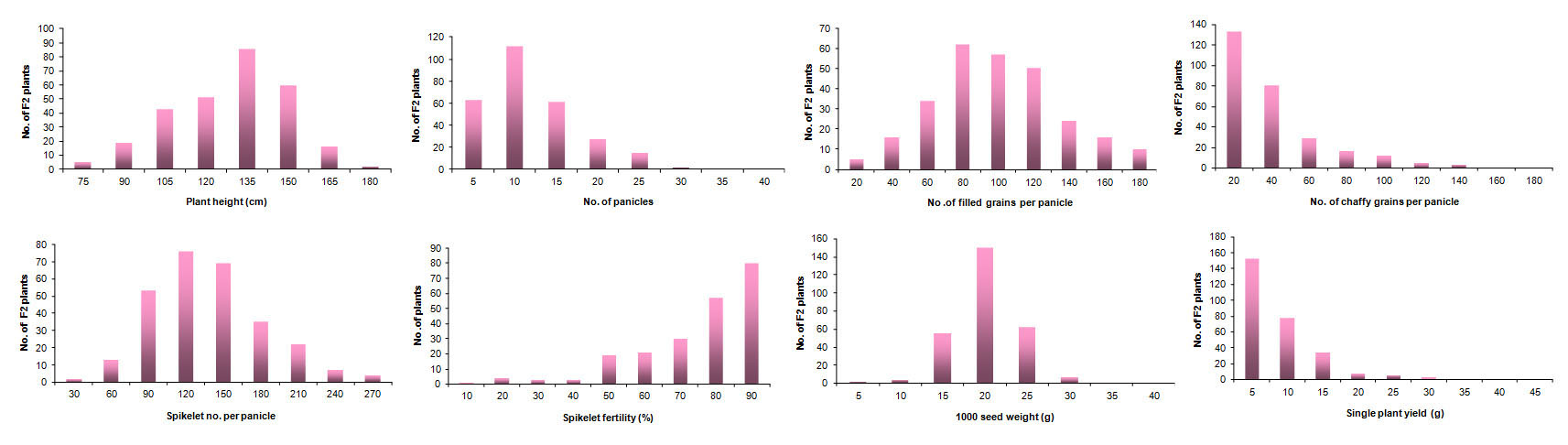

Supplement: Additional file 5: Figure S3. — Phenotypic distributions of agronomic traits in F3 population derived from a cross between Basmati370 and Jaya. B - Basmati370; J- Jaya; F1: Hybrid. (TIFF 2461 kb) [file 12870_2015_575_MOESM5_ESM.tif]
