# Supplementary material for: Discovery and mapping of genomic regions governing economically important traits of Basmati rice
Source: BMC Plant Biol. 2015 Aug 21;15:207. doi: 10.1186/s12870-015-0575-5 (PMC4546240; doi:10.1186/s12870-015-0575-5)
Supplement: Additional file 2: Table S1. — Transgressive segregants, heterosis, heterobeltiosis and inbreeding depression for 18 traits in the F2 population. (DOC 49 kb) [file 12870_2015_575_MOESM2_ESM.doc]

# Table S1 Transgressive segregants, heterosis, heterobeltiosis and inbreeding depression for 18 traits in the F_2_ population

| S.No. | Trait | F_2_ Mean  (n=181) | Transgressive segregants  (%) | Heterosis  (%) | Heterobeltiosis (%) | Inbreeding depression (%) |
| --- | --- | --- | --- | --- | --- | --- |
| 1 | Plant height (cm) | 112.85 | 49 | 20.39 | 4.76 | 6.15 |
| 2 | No. of panicles | 7.36 | 72 | 45.84 | 19.33 | 50.93 |
| 3 | Panicle length (cm) | 24.53 | 77 | 2.34 | -1.62 | 1.41 |
| 4 | Filled grains (no.) | 90.43 | 65 | 80.78 | 52.86 | 45.85 |
| 5 | Chaffy grains (no.) | 37.65 | 93 | 227.21 | 167.28 | -83.66 |
| 6 | Spikelet number* | 128.11 | 69 | 90.36 | 60.60 | 31.67 |
| 7 | Spikelet fertility* (%) | 71.55 | 100 | -5.11 | -5.39 | 19.66 |
| 8 | 1000 Seed weight (g) | 24.9 | 68 | 7.67 | -4.74 | -10.52 |
| 9 | Single plant yield (g) | 13.58 | 87 | 78.72 | 63.51 | 51.43 |
| 10 | Grain length (mm) | 6.09 | 44 | 0.32 | -3.85 | 2.40 |
| 11 | Grain breadth (mm) | 2.22 | 3 | 1.15 | -13.04 | -0.91 |
| 12 | Length-Breadth ratio* | 2.76 | 9 | -4.22 | -20.45 | 2.82 |
| 13 | Grain length after cooking (mm) | 14.48 | 33 | 24.9 | 3.31 | 7.18 |
| 14 | Elongation ratio* | 2.38 | 62 | 24.69 | 7.30 | 4.80 |
| 15 | Alkali spreading value | 5.75 | 28 | 0 | -14.29 | 4.17 |
| 16 | Amylose content (%) | 22.52 | 15 | -4.64 | -14.89 | 1.23 |
| 17 | Aroma | 4.66 | 0 | 0 | -44.44 | 6.80 |
| 18 | Chalkiness | 1.21 | 6 | 50 | 0.00 | 59.67 |

* Derived characters
