# Supplementary material for: Discovery and mapping of genomic regions governing economically important traits of Basmati rice
Source: BMC Plant Biol. 2015 Aug 21;15:207. doi: 10.1186/s12870-015-0575-5 (PMC4546240; doi:10.1186/s12870-015-0575-5)
Supplement: Additional file 3: Table S2. — Chi square values of microsatellite markers showing segregation distortion among F2 population of Basmati370/Jaya (DOC 154 kb) [file 12870_2015_575_MOESM3_ESM.doc]

**Table S2 Chi square values of microsatellite markers showing segregation distortion among F_2_ population of Basmati370/Jaya**

| S.No. | Marker | Chromosome | Chi square | Probability | Skewness |
| --- | --- | --- | --- | --- | --- |
| 1 | RM84 | 1 | 1159 | 0.00304 | Heterozygote |
| 2 | RM490 | 1 | 12.08 | 0.00238 | Basmati370 |
| 3 | RM259 | 1 | 22.63 | 0.00001 | Heterozygote |
| 4 | RM583 | 1 | 6.38 | 0.04121 | Basmati370 |
| 5 | RM579 | 1 | 13.24 | 0.00134 | Heterozygote |
| 6 | RM9 | 1 | 10.06 | 0.00655 | Heterozygote |
| 7 | RM306 | 1 | 16.81 | 0.00022 | Jaya |
| 8 | RM473A | 1 | 32.31 | 0.00000 | Heterozygote |
| 9 | RM128 | 1 | 18.91 | 0.00008 | Heterozygote |
| 10 | RM302 | 1 | 37.27 | 0.00000 | Basmati370 |
| 11 | RM212 | 1 | 18.93 | 0.00008 | Heterozygote |
| 12 | RM11968 | 1 | 22.00 | 0.00002 | Heterozygote |
| 13 | RM8278 | 1 | 12.75 | 0.00171 | Heterozygote |
| 14 | RM14 | 1 | 19.90 | 0.00005 | Heterozygote |
| 15 | RM5897 | 2 | 1185 | 0.00267 | Heterozygote |
| 16 | RM71 | 2 | 10.57 | 0.00506 | Heterozygote |
| 17 | RM327 | 2 | 12.42 | 0.00201 | Basmati370 |
| 18 | RM300 | 2 | 20.75 | 0.00003 | Heterozygote |
| 19 | RM475 | 2 | 13.71 | 0.00106 | Heterozygote |
| 20 | RM6318 | 2 | 15.74 | 0.00038 | Jaya |
| 21 | RM263 | 2 | 1151 | 0.00316 | Heterozygote |
| 22 | RM525 | 2 | 7.12 | 0.02841 | Jaya |
| 23 | RM318 | 2 | 14.02 | 0.00090 | Jaya |
| 24 | RM240 | 2 | 17.99 | 0.00012 | Heterozygote |
| 25 | RM112 | 2 | 1114 | 0.00382 | Heterozygote |
| 26 | RM138 | 2 | 6.64 | 0.03615 | Jaya |
| 27 | RM545 | 3 | 8.98 | 0.01124 | Heterozygote |
| 28 | RM517 | 3 | 12.45 | 0.00198 | Heterozygote |
| 29 | RM251 | 3 | 20.87 | 0.00003 | Heterozygote |
| 30 | RM282 | 3 | 17.27 | 0.00018 | Jaya |
| 31 | JL14 | 3 | 15.04 | 0.00054 | Jaya |
| 32 | RM353 | 3 | 18.00 | 0.00012 | Heterozygote |
| 33 | RM5864 | 3 | 19.73 | 0.00005 | Heterozygote |
| 34 | RM426 | 3 | 8.60 | 0.01360 | Jaya |
| 35 | RM168 | 3 | 17.17 | 0.00019 | Heterozygote |
| 36 | RM570 | 3 | 12.32 | 0.00211 | Heterozygote |
| 37 | RM565 | 3 | 26.24 | 0.00000 | Heterozygote |
| 38 | RM514 | 3 | 7.93 | 0.01901 | Heterozygote |
| 39 | RM85 | 3 | 10.76 | 0.00461 | Basmati370 |
| 40 | RM551 | 4 | 19.19 | 0.00007 | Heterozygote |
| 41 | RM518 | 4 | 13.24 | 0.00134 | Heterozygote |
| 42 | RM564 | 4 | 28.62 | 0.00000 | Heterozygote |
| 43 | RM252 | 4 | 8.41 | 0.01491 | Heterozygote |
| 44 | RM241 | 4 | 13.31 | 0.00129 | Jaya |
| 45 | RM127 | 4 | 6.64 | 0.03608 | Jaya |
| 46 | RM280 | 4 | 1139 | 0.00336 | Heterozygote |
| 47 | RM437 | 5 | 9.80 | 0.00746 | Jaya |
| 48 | RM289 | 5 | 2121 | 0.00002 | Heterozygote |
| S.No. | Marker | Chromosome | Chi square | Probability | Skewness |
| 49 | RM430 | 5 | 7.93 | 0.01901 | Jaya |
| 50 | RM18600 | 5 | 10.49 | 0.00526 | Jaya |
| 51 | RM161 | 5 | 6.18 | 0.04544 | Jaya |
| 52 | RM233 | 5 | 1184 | 0.00268 | Jaya |
| 53 | RM421 | 5 | 27.55 | 0.00000 | Heterozygote |
| 54 | MX4 | 6 | 10.38 | 0.00557 | Heterozygote |
| 55 | WXSSR | 6 | 13.02 | 0.00149 | Heterozygote |
| 56 | RM204 | 6 | 20.21 | 0.00004 | Heterozygote |
| 57 | RM225 | 6 | 13.21 | 0.00136 | Heterozygote |
| 58 | RM584 | 6 | 8.95 | 0.01139 | Heterozygote |
| 59 | RM539 | 6 | 8.22 | 0.01643 | Basmati370 |
| 60 | RM3 | 6 | 23.13 | 0.00001 | Heterozygote |
| 61 | RM162 | 6 | 7.16 | 0.02792 | Jaya |
| 62 | RM340 | 6 | 7.61 | 0.02222 | Jaya |
| 63 | RM481 | 7 | 8.11 | 0.01733 | Jaya |
| 64 | RM11 | 7 | 9.89 | 0.00712 | Jaya |
| 65 | RM336 | 7 | 16.30 | 0.00029 | Heterozygote |
| 66 | RM248 | 7 | 15.26 | 0.00049 | Heterozygote |
| 67 | RM408 | 8 | 26.69 | 0.00000 | Heterozygote |
| 68 | RM152 | 8 | 10.82 | 0.00447 | Heterozygote |
| 69 | RM310 | 8 | 3141 | 0.00000 | Heterozygote |
| 70 | RM547 | 8 | 32.86 | 0.00000 | Heterozygote |
| 71 | Fgr | 8 | 22.13 | 0.00002 | Heterozygote |
| 72 | RM72 | 8 | 1180 | 0.00273 | Jaya |
| 73 | RM44 | 8 | 15.10 | 0.00053 | Jaya |
| 74 | RM404 | 8 | 12.43 | 0.00200 | Jaya |
| 75 | RM483 | 8 | 7.12 | 0.02839 | Basmati370 |
| 76 | RM339 | 8 | 17.71 | 0.00014 | Jaya |
| 77 | RM42 | 8 | 27.74 | 0.00000 | Heterozygote |
| 78 | RM502 | 8 | 9.18 | 0.01013 | Jaya |
| 79 | RM464 | 9 | 53.70 | 0.00000 | Heterozygote |
| 80 | RM321 | 9 | 10.41 | 0.00550 | Basmati370 |
| 81 | RM566 | 9 | 7.58 | 0.02258 | Jaya |
| 82 | RM257 | 9 | 15.78 | 0.00038 | Heterozygote |
| 83 | RM242 | 9 | 16.50 | 0.00026 | Basmati370 |
| 84 | RM201 | 9 | 25.91 | 0.00000 | Basmati370 |
| 85 | RM107 | 9 | 15.72 | 0.00039 | Basmati370 |
| 86 | OSR28 | 9 | 2178 | 0.00002 | Basmati370 |
| 87 | RM474 | 10 | 14.93 | 0.00057 | Jaya |
| 88 | RM216 | 10 | 13.54 | 0.00115 | Jaya |
| 89 | RM258 | 10 | 9.89 | 0.00712 | Heterozygote |
| 90 | RM171 | 10 | 27.45 | 0.00000 | Heterozygote |
| 91 | RM228 | 10 | 6.65 | 0.03600 | Heterozygote |
| 92 | RM496 | 10 | 13.15 | 0.00140 | Jaya |
| 93 | RM590 | 10 | 20.16 | 0.00004 | Heterozygote |
| 94 | RM4B | 11 | 23.58 | 0.00001 | Jaya |
| 95 | RM1812 | 11 | 9.77 | 0.00755 | Heterozygote |
| 96 | RM202 | 11 | 13.99 | 0.00091 | Heterozygote |
| 97 | RM287 | 11 | 18.09 | 0.00012 | Heterozygote |
| 98 | RM235 | 12 | 56.35 | 0.00000 | Heterozygote |
