# Supplementary material for: Discovery and mapping of genomic regions governing economically important traits of Basmati rice
Source: BMC Plant Biol. 2015 Aug 21;15:207. doi: 10.1186/s12870-015-0575-5 (PMC4546240; doi:10.1186/s12870-015-0575-5)
Supplement: Additional file 6: Table S3. — Correlation coefficients among 18 traits of the RIL population derived from the cross of Basmati370 and Jaya. (DOC 60 kb) [file 12870_2015_575_MOESM6_ESM.doc]

**Table S**3. Correlation coefficients among 18 traits of the RIL population derived from the cross of Basmati370 and Jaya

| Trait | PH | NP | PL | FG | CG | SN | SF | SW | PY | GL | GB | LB | GLAC | ER | ASV | AC | ARM | CHK |
| --- | --- | --- | --- | --- | --- | --- | --- | --- | --- | --- | --- | --- | --- | --- | --- | --- | --- | --- |
| PH | 1 |  |  |  |  |  |  |  |  |  |  |  |  |  |  |  |  |  |
| NP | -0.108 | 1.000 |  |  |  |  |  |  |  |  |  |  |  |  |  |  |  |  |
| PL | **0.496*** | -0.080 | 1.000 |  |  |  |  |  |  |  |  |  |  |  |  |  |  |  |
| FG | 0.125 | 0.097 | 0.394 | 1.000 |  |  |  |  |  |  |  |  |  |  |  |  |  |  |
| CG | -0.023 | 0.005 | 0.283 | 0.011 | 1.000 |  |  |  |  |  |  |  |  |  |  |  |  |  |
| SN | 0.085 | 0.080 | **0.482*** | **0.799**** | **0.611**** | 1.000 |  |  |  |  |  |  |  |  |  |  |  |  |
| SF | 0.045 | 0.057 | -0.118 | 0.421 | **-0.880**** | -0.196 | 1.000 |  |  |  |  |  |  |  |  |  |  |  |
| SW | 0.064 | -0.174 | -0.084 | -0.190 | -0.143 | -0.237 | 0.050 | 1.000 |  |  |  |  |  |  |  |  |  |  |
| PY | 0.284 | **0.519*** | 0.270 | **0.505*** | -0.085 | 0.349 | 0.284 | 0.146 | 1.000 |  |  |  |  |  |  |  |  |  |
| GL | 0.164 | -0.004 | 0.043 | -0.202 | -0.189 | -0.274 | 0.128 | 0.382 | -0.009 | 1.000 |  |  |  |  |  |  |  |  |
| GB | 0.046 | -0.133 | 0.008 | -0.041 | 0.118 | 0.039 | -0.140 | 0.444 | 0.099 | -0.311 | 1.000 |  |  |  |  |  |  |  |
| LB | 0.046 | 0.109 | 0.005 | -0.075 | -0.170 | -0.161 | 0.155 | -0.160 | -0.077 | **0.717**** | **-0.873**** | 1.000 |  |  |  |  |  |  |
| GLAC | 0.007 | 0.032 | -0.009 | 0.010 | -0.110 | -0.059 | 0.111 | 0.066 | 0.047 | 0.040 | 0.095 | -0.052 | 1.000 |  |  |  |  |  |
| ER | 0.062 | 0.024 | 0.024 | 0.066 | -0.164 | -0.046 | 0.191 | 0.105 | 0.079 | 0.106 | 0.085 | -0.012 | **0.809**** | 1.000 |  |  |  |  |
| ASV | -0.014 | -0.026 | 0.074 | -0.013 | 0.070 | 0.032 | -0.084 | -0.199 | -0.097 | -0.106 | 0.033 | -0.068 | -0.034 | -0.049 | 1.000 |  |  |  |
| AC | 0.140 | -0.202 | 0.094 | 0.018 | 0.009 | 0.020 | -0.042 | -0.077 | -0.048 | -0.063 | 0.058 | -0.054 | 0.054 | 0.070 | -0.049 | 1.000 |  |  |
| ARM | -0.161 | 0.037 | -0.060 | -0.029 | 0.034 | -0.003 | -0.058 | -0.004 | -0.052 | -0.074 | 0.049 | -0.078 | -0.077 | -0.057 | -0.053 | 0.025 | 1.000 |  |
| CHK | 0.036 | 0.097 | -0.098 | -0.016 | -0.047 | -0.041 | 0.076 | -0.137 | -0.014 | 0.197 | -0.288 | 0.320 | -0.006 | -0.043 | -0.011 | 0.060 | 0.084 | 1.000 |

* Significant at p=0.05 ; ** Significant at p=0.01; For trait codes refer Table 1
