# Supplementary material for: Discovery and mapping of genomic regions governing economically important traits of Basmati rice
Source: BMC Plant Biol. 2015 Aug 21;15:207. doi: 10.1186/s12870-015-0575-5 (PMC4546240; doi:10.1186/s12870-015-0575-5)
Supplement: Additional file 7: Table S4. — Quantitative trait loci (QTLs) detected in F3 population of Basmati370/Jaya. (DOC 35 kb) [file 12870_2015_575_MOESM7_ESM.doc]

| Trait | Chr | QTL position | LFM | RFM | A | D | LOD | PVE(%) |
| --- | --- | --- | --- | --- | --- | --- | --- | --- |
| Plant height | 1 | 243 | RM302 | RM11968 | 8.0486 | 4.0637 | 4.3297 | 21.5541 |
| No. of Panicles | 1 | 77 | RM259 | RM579 | 0.5104 | 2.0865 | 6.2287 | 11.8598 |
|  | 5 | 129 | RM421 | RM459 | -0.2007 | 2.2637 | 3.6673 | 13.1927 |
| Chaffy grains (no.) | 1 | 73 | RM259 | RM579 | -8.5375 | -14.0631 | 4.2375 | 12.8035 |
|  | 3 | 194 | RM55 | RM448 | 9.1457 | 1.4615 | 2.6826 | 7.8573 |
| Sikelet number | 3 | 25 | RM130 | RM545 | -9.2942 | -18.0765 | 5.5484 | 18.6182 |
| Sikelet fertility | 2 | 193 | RM240 | RM112 | -9.3828 | -4.1558 | 5.4362 | 11.9135 |
| Single plant yield | 1 | 71 | RM259 | RM579 | -0.4032 | -1.2468 | 2.9535 | 8.5984 |
|  | 1 | 361 | RM8278 | RM582 | 1.5355 | -1.5522 | 3.6359 | 10.2049 |
|  | 9 | 209 | RM107 | RM566 | 1.5934 | -1.9178 | 3.6982 | 23.883 |

Table S4 Quantitative trait loci (QTLs) detected in F_3_ population of Basmati370/Jaya

PVE- Phenotypic variance explained by each QTL (%); Left (LFM) and right (RFM) flanking marker distance from the QTL (cM);Positive and negative values of additive effect indicates the increasing effect coming from the alleles of Basmati370 and Jaya respectively.
