# Supplementary material for: Discovery and mapping of genomic regions governing economically important traits of Basmati rice
Source: BMC Plant Biol. 2015 Aug 21;15:207. doi: 10.1186/s12870-015-0575-5 (PMC4546240; doi:10.1186/s12870-015-0575-5)
Supplement: Additional file 8: Table S5. — Quantitative trait loci (QTLs) detected in the RIL population derived from Basmati370/Jaya. (DOC 38 kb) [file 12870_2015_575_MOESM8_ESM.doc]

# Table S5 Quantitative trait loci (QTLs) detected RIL population derived from Basmati370/Jaya

| **SN** | **Trait** | **QTL** | **Chr.** | **Marker interval** | **LOD** | **A** | **D** | **PVE** |
| --- | --- | --- | --- | --- | --- | --- | --- | --- |
| 1 | Plant height (cm) | *qPH1.1* | 1 | RM302-RM11968 | 6.23 | 8.02 | -0.78 | 16.89 |
| 2 | Filled grains (no.) | *qFG1.1* | 1 | RM11968-RM14 | 3.65 | 25.02 | -22.32 | 12.29 |
| 3 | Single plant yield (g) | *qSPY9.1* | 9 | RM107-RM566 | 3.32 | 7.95 | -4.6 | 9.3 |
| 4 | Grain length (mm) | *qGL3.1* | 3 | RM353-JL14 | 11.36 | 0.59 | -0.210 | 35.36 |
| 5 |  | *qGL5.1* | 5 | RM430-RM18600 | 8.06 | 0.21 | 0.026 | 16.9 |
| 6 | Grain breadth (mm) | *qGB5.1* | 5 | RM430-RM18600 | 4.01 | -0.12 | 0.068 | 15.36 |
| 7 | Length-Breadth ratio | *qLB3.1* | 3 | RM353-JL14 | 5.32 | 0.26 | -0.354 | 25.88 |
| 8 |  | *qLB5.1* | 5 | RM430-RM18600 | 4.9 | 0.39 | -0.06 | 39.66 |
| 9 | Elongation ratio | *qER5.1* | 5 | RM430-RM18600 | 4.21 | -0.15 | 0.09 | 17.39 |
| 10 | Alkali spreading value | *qASV6.1* | 6 | RM276-RM527 | 27.33 | -1.29 | 0.36 | 73.52 |
| 11 | Aroma | *qARM8.1* | 8 | RM404-RM483 | 5.06 | 2.6 | 0.45 | 18.16 |
| 12 | Chalkiness | *qCHK4.1* | 4 | RM564-RM348 | 3.6 | 1.96 | -0.136 | 48.02 |

PVE- Phenotypic variance explained by each QTL (%);A- Additive effect; D-Dominance effect; Positive and negative values of additive effect indicates the increasing effect coming from the alleles of Basmati370 and Jaya respectively.
