# Supplementary material for: Discovery and mapping of genomic regions governing economically important traits of Basmati rice
Source: BMC Plant Biol. 2015 Aug 21;15:207. doi: 10.1186/s12870-015-0575-5 (PMC4546240; doi:10.1186/s12870-015-0575-5)
Supplement: Additional file 9: Table S6 — The genes with non-synonymous SNPs in the QTL for filled grain qFG1.1. (RM11968-RM14). (DOC 65 kb) [file 12870_2015_575_MOESM9_ESM.doc]

| Table S6 The genes with non-synonymous SNPs in the QTL for filled grain qFG1.1 (RM11968-RM14) | |  |  |  |
| --- | --- | --- | --- | --- |
| **Gene** | **Function** | **SNP Position** | **Non-Synonymous SNP** | **Amino acid change** |
| LOC_Os01g68990 | retrotransposon, putative, centromere-specific, expressed | 40082323 | Tgg/Cgg | W30R |
| LOC_Os01g69000 | retrotransposon protein, putative, unclassified | 40084411 | aaC/aaG | N6K |
| LOC_Os01g69800 | transposon protein, putative, CACTA, En/Spm sub-class, expressed | 40303442 | Cgg/Tgg | R10W |
| LOC_Os01g69800 | transposon protein, putative, CACTA, En/Spm sub-class, expressed | 40304057 | aCt/aGt | T178S |
| LOC_Os01g69850 | OsMADS65 - MADS-box family gene with MIKC* type-box, expressed | 40362268 | gGt/gAt | G120D |
| LOC_Os01g69850 | OsMADS65 - MADS-box family gene with MIKC* type-box, expressed | 40362776 | tCc/tAc | S152Y |
| LOC_Os01g69990 | GYF domain containing protein, putative, expressed | 40484299 | Ttg/Gtg | L1405V |
| LOC_Os01g69990 | GYF domain containing protein, putative, expressed | 40485325 | Ttg/Atg | L1131M |
| LOC_Os01g69990 | GYF domain containing protein, putative, expressed | 40486656 | gGa/gCa | G687A |
| LOC_Os01g69990 | GYF domain containing protein, putative, expressed | 40486906 | Gca/Aca | A639T |
| LOC_Os01g69990 | GYF domain containing protein, putative, expressed | 40488407 | Act/Cct | T210P |
| LOC_Os01g70020 | DEK C terminal domain containing protein, expressed | 40505751 | gAt/gGt | D265G |
| LOC_Os01g70040 | transposon protein, putative, CACTA, En/Spm sub-class | 40521539 | aaA/aaC | K681N |
| LOC_Os01g70140 | ubiquitin-conjugating enzyme E2-22 kDa, putative, expressed | 40596478 | aAt/aGt | N152S |
| LOC_Os01g70220 | histone-lysine N-methyltransferase, putative, expressed | 40654878 | tTg/tCg | L33S |
| LOC_Os01g70220 | histone-lysine N-methyltransferase, putative, expressed | 40659706 | tCg/tTg | S366L |
| LOC_Os01g70250 | heat shock protein DnaJ, putative, expressed | 40675807 | tTg/tCg | L572S |
| LOC_Os01g70250 | heat shock protein DnaJ, putative, expressed | 40675963 | gTt/gCt | V520A |
| LOC_Os01g70250 | heat shock protein DnaJ, putative, expressed | 40676767 | tCg/tTg | S252L |
| LOC_Os01g70270 | auxin response factor, putative, expressed | 40698889 | cGa/cAa | R530Q |
| LOC_Os01g70450 | expressed protein | 40804256 | Aat/Gat | N97D |
| LOC_Os01g70480 | expressed protein | 40823438 | Tac/Cac | Y129H |
| LOC_Os01g70480 | expressed protein | 40823642 | Gtt/Att | V61 |
| LOC_Os01g70490 | potassium transporter, putative, expressed | 40828184 | Att/Gtt | 396V |
| LOC_Os01g70580 | thiamin pyrophosphokinase 1, putative, expressed | 40868955 | agG/agT | R249S |
| LOC_Os01g70600 | DUF567 domain containing protein, putative, expressed | 40880032 | Atc/Ttc | 86F |
| LOC_Os01g70600 | DUF567 domain containing protein, putative, expressed | 40880987 | Ggc/Agc | G245S |
| LOC_Os01g70670 | BTBM1 - Bric-a-Brac, Tramtrack, Broad Complex BTB domain with Meprin and TRAF Homology MATH domain, expressed | 40917846 | Tct/Cct | S250P |
| LOC_Os01g70810 | homeobox domain containing protein, expressed | 40989323 | aTt/aCt | 500T |
| LOC_Os01g70810 | homeobox domain containing protein, expressed | 40991178 | Act/Gct | T696A |
| LOC_Os01g70810 | homeobox domain containing protein, expressed | 40991846 | gTt/gCt | V891A |
| LOC_Os01g70810 | homeobox domain containing protein, expressed | 40992386 | tAt/tCt | Y1071S |
| LOC_Os01g71114 | NBS-LRR disease resistance protein, putative, expressed | 41160827 | cCt/cGt | P517R |
| LOC_Os01g71114 | NBS-LRR disease resistance protein, putative, expressed | 41162266 | Gca/Tca | A397S |
| LOC_Os01g71130 | xylanase inhibitor 725OS, putative, expressed | 41166316 | Cca/Gca | P4A |
| LOC_Os01g71180 | pentatricopeptide repeat protein PPR1106-17, putative, expressed | 41185401 | atA/atG | 534M |
| LOC_Os01g71270 | eukaryotic peptide chain release factor subunit 1-1, putative, expressed | 41242605 | Gtc/Ttc | V47F |
| LOC_Os01g71630 | expressed protein | 41517612 | Gtg/Atg | V119M |
| LOC_Os01g71630 | expressed protein | 41518960 | aCg/aAg | T322K |
| LOC_Os01g71840 | retrotransposon protein, putative, unclassified, expressed | 41613234 | Ggc/Agc | G1641S |
| LOC_Os01g71960 | endonuclease, putative, expressed | 41694812 | atA/atG | 333M |
| LOC_Os01g71960 | endonuclease, putative, expressed | 41695168 | aTt/aCt | 374T |
| LOC_Os01g71960 | endonuclease, putative, expressed | 41697771 | cTg/cCg | L570P |
| LOC_Os01g72110 | transposon protein, putative, unclassified, expressed | 41831044 | aAc/aCc | N484T |
| LOC_Os01g72400 | retrotransposon protein, putative, Ty1-copia subclass, expressed | 41994316 | atG/atA | M710 |
| LOC_Os01g72400 | retrotransposon protein, putative, Ty1-copia subclass, expressed | 41994323 | tCt/tTt | S708F |
| LOC_Os01g72990 | expressed protein | 42338439 | Ata/Gta | 778V |
| LOC_Os01g73344 | retrotransposon protein, putative, Ty3-gypsy subclass | 42512837 | Cac/Tac | H377Y |
