# Supplementary material for: Discovery and mapping of genomic regions governing economically important traits of Basmati rice
Source: BMC Plant Biol. 2015 Aug 21;15:207. doi: 10.1186/s12870-015-0575-5 (PMC4546240; doi:10.1186/s12870-015-0575-5)
Supplement: Additional file 10: Table S7 — The genes with non-synonymous SNPs in the QTL for chalkiness qCHK4.1. (RM564-RM348) (DOC 168 kb) [file 12870_2015_575_MOESM10_ESM.doc]

| Table S7 The genes with non-synonymous SNPs in the QTL for chalkiness qCHK4.1 (RM564-RM348) | |  |  |  |
| --- | --- | --- | --- | --- |
|  |  |  |  |  |
| **Gene** | **Function** | **SNP Position** | **Non-Synonymous SNP** | **Amino acid change** |
| LOC_Os04g35160 | CorA-like magnesium transporter protein, putative, expressed | 21370161 | Acc/Tcc | T216S |
| LOC_Os04g35230 | expressed protein | 21411375 | Cgg/Tgg | R51W |
| LOC_Os04g35420 | helicase conserved C-terminal domain containing protein, expressed | 21550286 | Act/Tct | T756S |
| LOC_Os04g35510 | CENP-E-like kinetochore protein, putative, expressed | 21610862 | aGc/aTc | S122 |
| LOC_Os04g35940 | OsFBX135 - F-box domain containing protein, expressed | 21913302 | Gtg/Ctg | V156L |
| LOC_Os04g37790 | expressed protein | 22468483 | cGc/cAc | R41H |
| LOC_Os04g37800 | hypothetical protein | 22471270 | Act/Cct | T61P |
| LOC_Os04g37830 | retrotransposon protein, putative, unclassified, expressed | 22489648 | Agc/Ggc | S217G |
| LOC_Os04g39990 | retrotransposon protein, putative, unclassified, expressed | 23801528 | Atc/Gtc | 249V |
| LOC_Os04g40740 | kelch repeat protein, putative, expressed | 24181079 | aGg/aAg | R361K |
| LOC_Os04g40820 | retrotransposon protein, putative, unclassified, expressed | 24223549 | aGt/aTt | S28 |
| LOC_Os04g41840 | transposon protein, putative, unclassified, expressed | 24789258 | Cct/Tct | P1317S |
| LOC_Os04g42080 | expressed protein | 24913470 | Gat/Aat | D26N |
| LOC_Os04g42150 | hypothetical protein | 24959110 | aCa/aTa | T119 |
| LOC_Os04g42250 | transferase family protein, putative, expressed | 24998494 | Cgc/Tgc | R242C |
| LOC_Os04g42320 | AT hook motif family protein, expressed | 25042487 | tCt/tTt | S350F |
| LOC_Os04g42760 | sialyltransferase family domain containing protein, expressed | 25308323 | Aac/Gac | N372D |
| LOC_Os04g42770 | expressed protein | 25316548 | aCt/aTt | T367 |
| LOC_Os04g44370 | retrotransposon protein, putative, unclassified, expressed | 26270254 | Aac/Gac | N1216D |
| LOC_Os04g44370 | retrotransposon protein, putative, unclassified, expressed | 26270700 | gGg/gAg | G1067E |
| LOC_Os04g44370 | retrotransposon protein, putative, unclassified, expressed | 26271466 | Tgc/Cgc | C862R |
| LOC_Os04g44370 | retrotransposon protein, putative, unclassified, expressed | 26271796 | Ggg/Agg | G752R |
| LOC_Os04g44370 | retrotransposon protein, putative, unclassified, expressed | 26271945 | tCg/tTg | S702L |
| LOC_Os04g45420 | retrotransposon protein, putative, unclassified, expressed | 26863114 | Atc/Gtc | 2469V |
| LOC_Os04g45420 | retrotransposon protein, putative, unclassified, expressed | 26864871 | cTg/cCg | L2039P |
| LOC_Os04g45420 | retrotransposon protein, putative, unclassified, expressed | 26865028 | Gtc/Atc | V1987 |
| LOC_Os04g45420 | retrotransposon protein, putative, unclassified, expressed | 26865160 | Gca/Aca | A1943T |
| LOC_Os04g45420 | retrotransposon protein, putative, unclassified, expressed | 26865312 | gCt/gAt | A1892D |
| LOC_Os04g45420 | retrotransposon protein, putative, unclassified, expressed | 26865331 | Aac/Gac | N1886D |
| LOC_Os04g45420 | retrotransposon protein, putative, unclassified, expressed | 26865600 | cTt/cAt | L1796H |
| LOC_Os04g45420 | retrotransposon protein, putative, unclassified, expressed | 26865958 | Ggt/Agt | G1677S |
| LOC_Os04g45420 | retrotransposon protein, putative, unclassified, expressed | 26866959 | cAc/cGc | H1523R |
| LOC_Os04g45420 | retrotransposon protein, putative, unclassified, expressed | 26867119 | Gtg/Atg | V1470M |
| LOC_Os04g45420 | retrotransposon protein, putative, unclassified, expressed | 26867571 | aGa/aAa | R1319K |
| LOC_Os04g45420 | retrotransposon protein, putative, unclassified, expressed | 26867583 | tCa/tTa | S1315L |
| LOC_Os04g45420 | retrotransposon protein, putative, unclassified, expressed | 26867638 | Gct/Act | A1297T |
| LOC_Os04g45420 | retrotransposon protein, putative, unclassified, expressed | 26867706 | aCt/aAt | T1274N |
| LOC_Os04g45420 | retrotransposon protein, putative, unclassified, expressed | 26867709 | cCa/cTa | P1273L |
| LOC_Os04g45420 | retrotransposon protein, putative, unclassified, expressed | 26867836 | Aag/Gag | K1231E |
| LOC_Os04g45420 | retrotransposon protein, putative, unclassified, expressed | 26869535 | aaC/aaA | N717K |
| LOC_Os04g45420 | retrotransposon protein, putative, unclassified, expressed | 26869864 | Gca/Cca | A608P |
| LOC_Os04g45860 | transposon protein, putative, unclassified, expressed | 27155597 | aCa/aGa | T368R |
| LOC_Os04g47080 | anthocyanin regulatory Lc protein, putative, expressed | 27954127 | gGc/gAc | G50D |
| LOC_Os04g47590 | niemann-Pick C1 protein precursor, putative, expressed | 28239241 | gCt/gTt | A76V |
| LOC_Os04g48300 | hypothetical protein | 28787154 | cGg/cAg | R117Q |
| LOC_Os04g48670 | retrotransposon protein, putative, Ty3-gypsy subclass, expressed | 29015723 | Gca/Tca | A281S |
| LOC_Os04g48670 | retrotransposon protein, putative, Ty3-gypsy subclass, expressed | 29015933 | Agg/Ggg | R211G |
| LOC_Os04g48770 | ubiquitin fusion degradation protein, putative, expressed | 29092013 | Gtt/Ttt | V158F |
| LOC_Os04g49240 | expressed protein | 29385962 | Aca/Gca | T55A |
| LOC_Os04g49300 | retrotransposon protein, putative, LINE subclass, expressed | 29424206 | aTa/aCa | 67T |
| LOC_Os04g49680 | DUF581 domain containing protein, expressed | 29624766 | Gcg/Acg | A97T |
| LOC_Os04g49790 | retrotransposon protein, putative, Ty1-copia subclass, expressed | 29683488 | cGt/cAt | R843H |
| LOC_Os04g49790 | retrotransposon protein, putative, Ty1-copia subclass, expressed | 29684160 | Cgc/Tgc | R691C |
| LOC_Os04g49790 | retrotransposon protein, putative, Ty1-copia subclass, expressed | 29684972 | cCa/cTa | P500L |
| LOC_Os04g49790 | retrotransposon protein, putative, Ty1-copia subclass, expressed | 29687497 | Aca/Cca | T158P |
| LOC_Os04g49860 | DNA2 - Putative DNA replication helicase 2 protein, expressed | 29721352 | caT/caA | H363Q |
| LOC_Os04g49860 | DNA2 - Putative DNA replication helicase 2 protein, expressed | 29729200 | Aca/Tca | T1212S |
| LOC_Os04g49900 | ABC transporter family protein, putative, expressed | 29757258 | gAc/gGc | D288G |
| LOC_Os04g49900 | ABC transporter family protein, putative, expressed | 29757584 | Atc/Gtc | 397V |
| LOC_Os04g49900 | ABC transporter family protein, putative, expressed | 29758121 | Ttt/Ctt | F576L |
| LOC_Os04g49900 | ABC transporter family protein, putative, expressed | 29761810 | Acc/Gcc | T1280A |
| LOC_Os04g49930 | sensitivity to red light reduced protein 1, putative, expressed | 29776990 | cGt/cAt | R801H |
| LOC_Os04g49930 | sensitivity to red light reduced protein 1, putative, expressed | 29778118 | Tgc/Cgc | C490R |
| LOC_Os04g49930 | sensitivity to red light reduced protein 1, putative, expressed | 29781024 | tGt/tCt | C331S |
| LOC_Os04g50192 | retrotransposon protein, putative, LINE subclass, expressed | 29948225 | tCc/tTc | S666F |
| OC_Os04g51250 | expressed protein | 30368835 | tgT/tgG | C6WL |
| LOC_Os04g51500 | expressed protein | 30511492 | gGt/gAt | G76D |
| LOC_Os04g51500 | expressed protein | 30511760 | aTt/aCt | 19T |
| LOC_Os04g51740 | retrotransposon protein, putative, Ty3-gypsy subclass | 30658271 | gCa/gTa | A1463V |
| LOC_Os04g51740 | retrotransposon protein, putative, Ty3-gypsy subclass | 30658280 | aAg/aGg | K1460R |
| LOC_Os04g51740 | retrotransposon protein, putative, Ty3-gypsy subclass | 30659133 | Gtc/Atc | V1176 |
| LOC_Os04g51740 | retrotransposon protein, putative, Ty3-gypsy subclass | 30660834 | Act/Gct | T609A |
| LOC_Os04g51794 | DNA binding protein, putative, expressed | 30691873 | Ata/Tta | 326L |
| LOC_Os04g52074 | expressed protein | 30929924 | Tat/Gat | Y373D |
| LOC_Os04g52230 | ent-kaurene synthase, chloroplast precursor, putative, expressed | 31030202 | aCc/aTc | T630 |
| LOC_Os04g52460 | retrotransposon protein, putative, unclassified, expressed | 31194609 | Act/Gct | T1337A |
| LOC_Os04g52460 | retrotransposon protein, putative, unclassified, expressed | 31194970 | gAa/gGa | E1356G |
| LOC_Os04g52520 | APO, putative, expressed | 31226730 | aTc/aCc | 19T |
| LOC_Os04g52540 | retrotransposon protein, putative, unclassified, expressed | 31240150 | Gct/Tct | A183S |
| LOC_Os04g52540 | retrotransposon protein, putative, unclassified, expressed | 31240217 | gCc/gAc | A205D |
| LOC_Os04g52590 | protein kinase domain containing protein, expressed | 31264557 | gGt/gAt | G721D |
| LOC_Os04g52590 | protein kinase domain containing protein, expressed | 31264880 | Tgt/Cgt | C640R |
| LOC_Os04g52590 | protein kinase domain containing protein, expressed | 31264928 | Aaa/Gaa | K624E |
| LOC_Os04g52590 | protein kinase domain containing protein, expressed | 31266543 | aaA/aaT | K403N |
| LOC_Os04g52590 | protein kinase domain containing protein, expressed | 31268322 | tTg/tCg | L205S |
| LOC_Os04g52614 | SHR5-receptor-like kinase, putative, expressed | 31301607 | Aca/Gca | T644A |
| LOC_Os04g52614 | SHR5-receptor-like kinase, putative, expressed | 31301932 | caT/caG | H563Q |
| LOC_Os04g52614 | SHR5-receptor-like kinase, putative, expressed | 31302587 | aAt/aGt | N392S |
| LOC_Os04g52614 | SHR5-receptor-like kinase, putative, expressed | 31302601 | gaC/gaA | D387E |
| LOC_Os04g52614 | SHR5-receptor-like kinase, putative, expressed | 31302654 | Aat/Tat | N370Y |
| LOC_Os04g52614 | SHR5-receptor-like kinase, putative, expressed | 31302668 | cAa/cGa | Q365R |
| LOC_Os04g52614 | SHR5-receptor-like kinase, putative, expressed | 31302734 | tGt/tCt | C343S |
| LOC_Os04g52630 | leucine-rich repeat-containing protein kinase family protein, putative, expressed | 31311675 | Att/Gtt | 429V |
| LOC_Os04g52640 | SHR5-receptor-like kinase, putative, expressed | 31322465 | aGa/aAa | R436K |
| LOC_Os04g52640 | SHR5-receptor-like kinase, putative, expressed | 31323216 | gGt/gAt | G274D |
| LOC_Os04g52640 | SHR5-receptor-like kinase, putative, expressed | 31325623 | tTc/tAc | F185Y |
| LOC_Os04g52670 | OsSAUR21 - Auxin-responsive SAUR gene family member, expressed | 31350359 | Act/Gct | T122A |
| LOC_Os04g52690 | AAA family ATPase, putative, expressed | 31364717 | Aca/Gca | T917A |
| LOC_Os04g52725 | PPR repeat domain containing protein, putative, expressed | 31399508 | Tgt/Ggt | C235G |
| LOC_Os04g52940 | SIT4 phosphatase-associated protein domain containing protein, expressed | 31535848 | aTa/aCa | 163T |
| LOC_Os04g52960 | nucleolin, putative, expressed | 31547177 | tTa/tCa | L150S |
| LOC_Os04g52960 | nucleolin, putative, expressed | 31549328 | cCg/cTg | P430L |
| LOC_Os04g52970 | NBS-LRR disease resistance protein, putative, expressed | 31556683 | agT/agG | S1032R |
| LOC_Os04g53030 | NBS-LRR disease resistance protein, putative, expressed | 31581603 | aCa/aAa | T1120K |
| LOC_Os04g53030 | NBS-LRR disease resistance protein, putative, expressed | 31581648 | tCt/tAt | S1135Y |
| LOC_Os04g53030 | NBS-LRR disease resistance protein, putative, expressed | 31581963 | aTa/aCa | 1240T |
| LOC_Os04g53030 | NBS-LRR disease resistance protein, putative, expressed | 31582128 | cTc/cGc | L1295R |
| LOC_Os04g53030 | NBS-LRR disease resistance protein, putative, expressed | 31582217 | Cat/Aat | H1325N |
| LOC_Os04g53030 | NBS-LRR disease resistance protein, putative, expressed | 31582472 | Tgc/Ggc | C1410G |
| LOC_Os04g53030 | NBS-LRR disease resistance protein, putative, expressed | 31582584 | cAa/cGa | Q1447R |
| LOC_Os04g53040 | retrotransposon protein, putative, unclassified, expressed | 31594897 | Acc/Gcc | T1295A |
| LOC_Os04g53190 | CPuORF12 - conserved peptide uORF-containing transcript, expressed | 31670426 | gTt/gCt | V237A |
| LOC_Os04g53200 | exonuclease, putative, expressed | 31682752 | Atc/Gtc | 125V |
| LOC_Os04g53250 | polyphenol oxidase protein, putative, expressed | 31722162 | Agc/Ggc | S84G |
| LOC_Os04g53310 | soluble starch synthase 3, chloroplast precursor, putative, expressed | 31758185 | aaA/aaC | K207N |
| LOC_Os04g53350 | expressed protein | 31769832 | Gat/Tat | D534Y |
| LOC_Os04g53360 | expressed protein | 31779936 | gGa/gAa | G512E |
| LOC_Os04g53380 | expressed protein | 31793461 | aTt/aCt | 2039T |
| LOC_Os04g53380 | expressed protein | 31794052 | Gta/Ata | V1887 |
| LOC_Os04g53380 | expressed protein | 31794832 | Tgc/Cgc | C1627R |
| LOC_Os04g53380 | expressed protein | 31796817 | aCc/aTc | T965 |
| LOC_Os04g53380 | expressed protein | 31798144 | tTt/tGt | F805C |
| LOC_Os04g53390 | MrBTB2 - Bric-a-Brac, Tramtrack, Broad Complex BTB domain with Meprin and TRAF Homology MATH-related domain, expressed | 31801997 | Gag/Cag | E22Q |
| LOC_Os04g53390 | MrBTB2 - Bric-a-Brac, Tramtrack, Broad Complex BTB domain with Meprin and TRAF Homology MATH-related domain, expressed | 31802898 | tTc/tCc | F197S |
| LOC_Os04g53496 | NBS-LRR disease resistance protein, putative, expressed | 31857708 | Acc/Tcc | T1094S |
| LOC_Os04g53496 | NBS-LRR disease resistance protein, putative, expressed | 31859271 | Aag/Gag | K1502E |
| LOC_Os04g53496 | NBS-LRR disease resistance protein, putative, expressed | 31859346 | Gga/Aga | G1527R |
| LOC_Os04g53496 | NBS-LRR disease resistance protein, putative, expressed | 31860211 | gGt/gTt | G1577V |
| LOC_Os04g53496 | NBS-LRR disease resistance protein, putative, expressed | 31862159 | cGg/cAg | R1766Q |
| LOC_Os04g54020 | receptor-like kinase, putative, expressed | 32200210 | Tat/Cat | Y586H |
| LOC_Os04g54050 | retrotransposon protein, putative, unclassified | 32211783 | tCg/tTg | S250L |
| LOC_Os04g54780 | retrotransposon protein, putative, Ty3-gypsy subclass, expressed | 32570963 | gaA/gaC | E325D |
| LOC_Os04g54780 | retrotransposon protein, putative, Ty3-gypsy subclass, expressed | 32571507 | cTa/cCa | L470P |
| LOC_Os04g54820 | receptor-like kinase, putative, expressed | 32600938 | Ggt/Agt | G19S |
| LOC_Os04g54820 | receptor-like kinase, putative, expressed | 32601615 | cGt/cAt | R139H |
| LOC_Os04g54830 | expressed protein | 32608416 | gAc/gCc | D637A |
| LOC_Os04g54850 | pectinesterase, putative, expressed | 32627169 | tCc/tTc | S442F |
| LOC_Os04g55030 | cullin, putative, expressed | 32728736 | Gca/Aca | A126T |
| LOC_Os04g55140 | retrotransposon protein, putative, Ty1-copia subclass, expressed | 32784993 | Acg/Gcg | T2AL |
| LOC_Os04g55190 | SPOC domain containing protein, putative, expressed | 32808506 | gTc/gGc | V813G |
| LOC_Os04g55230 | tetratricopeptide repeat domain containing protein, putative, expressed | 32840515 | Gca/Aca | A274T |
| LOC_Os04g55230 | tetratricopeptide repeat domain containing protein, putative, expressed | 32840995 | cTa/cCa | L399P |
| LOC_Os04g55300 | hypothetical protein | 32872108 | Gtg/Atg | V56M |
| LOC_Os04g55360 | ubiquitin carboxyl-terminal hydrolase domain containing protein, expressed | 32933788 | gaA/gaC | E340D |
| LOC_Os04g55360 | ubiquitin carboxyl-terminal hydrolase domain containing protein, expressed | 32933885 | aTg/aCg | M308T |
| LOC_Os04g55370 | plant protein of unknown function domain containing protein, expressed | 32940893 | Tca/Gca | S312A |
| LOC_Os04g55440 | Cf-4A protein, putative, expressed | 32974922 | Tcc/Ccc | S27P |
| LOC_Os04g55440 | Cf-4A protein, putative, expressed | 32975130 | cTc/cAc | L96H |
| LOC_Os04g55550 | expressed protein | 33053060 | Aaa/Caa | K9QL |
| LOC_Os04g55570 | plant protein of unknown function DUF869 domain containing protein, expressed | 33084202 | gCg/gTg | A449V |
| LOC_Os04g55570 | plant protein of unknown function DUF869 domain containing protein, expressed | 33086134 | aGg/aAg | R909K |
| LOC_Os04g55630 | expressed protein | 33115378 | tCg/tTg | S149L |
| LOC_Os04g55700 | exonuclease, putative, expressed | 33150357 | gCa/gTa | A457V |
| LOC_Os04g55700 | exonuclease, putative, expressed | 33150393 | aAg/aTg | K445M |
| LOC_Os04g55740 | peroxidase precursor, putative, expressed | 33181178 | gAc/gCc | D48A |
| LOC_Os04g55770 | expressed protein | 33202284 | Gat/Aat | D124N |
| LOC_Os04g55810 | expressed protein | 33226069 | Ccg/Tcg | P454S |
| LOC_Os04g55810 | expressed protein | 33226087 | Aca/Gca | T448A |
| LOC_Os04g55810 | expressed protein | 33226125 | tAt/tCt | Y435S |
| LOC_Os04g55810 | expressed protein | 33227332 | cTt/cCt | L309P |
| LOC_Os04g55810 | expressed protein | 33228709 | aAt/aCt | N224T |
